# The Impact of Long Non-Coding RNAs in the Pathogenesis of Hepatocellular Carcinoma

**DOI:** 10.3389/fonc.2021.649107

**Published:** 2021-04-21

**Authors:** Soudeh Ghafouri-Fard, Mahdi Gholipour, Bashdar Mahmud Hussen, Mohammad Taheri

**Affiliations:** ^1^ Urogenital Stem Cell Research Center, Shahid Beheshti University of Medical Sciences, Tehran, Iran; ^2^ Department of Medical Genetics, Shahid Beheshti University of Medical Sciences, Tehran, Iran; ^3^ Pharmacognosy Department, College of Pharmacy, Hawler Medical University, Erbil, Iraq; ^4^ Urology and Nephrology Research Center, Shahid Beheshti University of Medical Sciences, Tehran, Iran

**Keywords:** lncRNA, biomarker, hepatocellular carcinoma, expression, polymorphism

## Abstract

Hepatocellular carcinoma (HCC) is among the utmost deadly human malignancies. This type of cancer has been associated with several environmental, viral, and lifestyle risk factors. Among the epigenetic factors which contribute in the pathogenesis of HCC is dysregulation of long non-coding RNAs (lncRNAs). These transcripts modulate expression of several tumor suppressor genes and oncogenes and alter the activity of cancer-related signaling axes. Several lncRNAs such as NEAT1, MALAT1, ANRIL, and SNHG1 have been up-regulated in HCC samples. On the other hand, a number of so-called tumor suppressor lncRNAs namely CASS2 and MEG3 are down-regulated in HCC. The interaction between lncRNAs and miRNAs regulate expression of a number of mRNA coding genes which are involved in the pathogenesis of HCC. H19/miR-15b/CDC42, H19/miR-326/TWIST1, NEAT1/miR-485/STAT3, MALAT1/miR-124-3p/Slug, MALAT1/miR-195/EGFR, MALAT1/miR-22/SNAI1, and ANRIL/miR-144/PBX3 axes are among functional axes in the pathobiology of HCC. Some genetic polymorphisms within non-coding regions of the genome have been associated with risk of HCC in certain populations. In the current paper, we describe the recent finding about the impact of lncRNAs in HCC.

## Introduction

Liver cancer is among the most lethal malignancies among both sexes. More than 8% of cancer-related mortalities are due to this type of cancer (1). Hepatocellular carcinoma (HCC) includes more than 75% of the primary liver neoplasms (1). Several factors have been related with elevated risk of HCC among them are chronic infection with hepatitis B virus (HBV) B or hepatitis C virus (HCV), dietary exposure with aflatoxin, excessive alcohol use, obesity, and smoking (2). The cirrhosis-induced carcinogenic alterations have been detected in 90% of HCC patients (3). High throughput sequencing methods have shown the occurrence of several genetic changes in the HCC samples (4) among the early events are inactivating mutations in insulin-like growth factor 2 receptor (5). Catenin Beta 1 (CTNNB1) and Tumor Protein P53 (TP53) are the utmost recurrently mutated oncogene and tumor suppressor gene in HCC, respectively (4). In addition to these somatic mutations, several epigenetic factors partake in the evolution of HCC. Such involvement is further highlighted by the fact that liver is an organs that is continuously adapting to extremely various environmental factors (6). Non-coding RNAs are among epigenetic elements that contribute in the pathogenesis of HCC. Long non-coding RNAs (lncRNAs) can affect expression of genes *via* diverse mechanisms including recruitment of regulatory protein complexes, acting as a decoy, changing genome organization and modulating the distribution of posttranslational modifications (7). These transcripts have sizes longer than 200 nucleotides and are comparable with mRNAs in the terms of chromatin state of genome loci, their transcription by RNA polymerase II, polyadenylation, 5’ capping and being spliced, yet they do not produce large-sized polypeptides (8). However, there are several reports demonstrating the presence of stable, functional micropeptides being translated from lncRNAs (9). Several lines of evidence indicates that these transcripts contribute in the pathophysiology of HCC (10). In the present manuscript, we review the current knowledge about the partake of lncRNAs in the pathogenesis of HCC.

## Up-regulated lncRNAs in HCC

**Figure 1:**
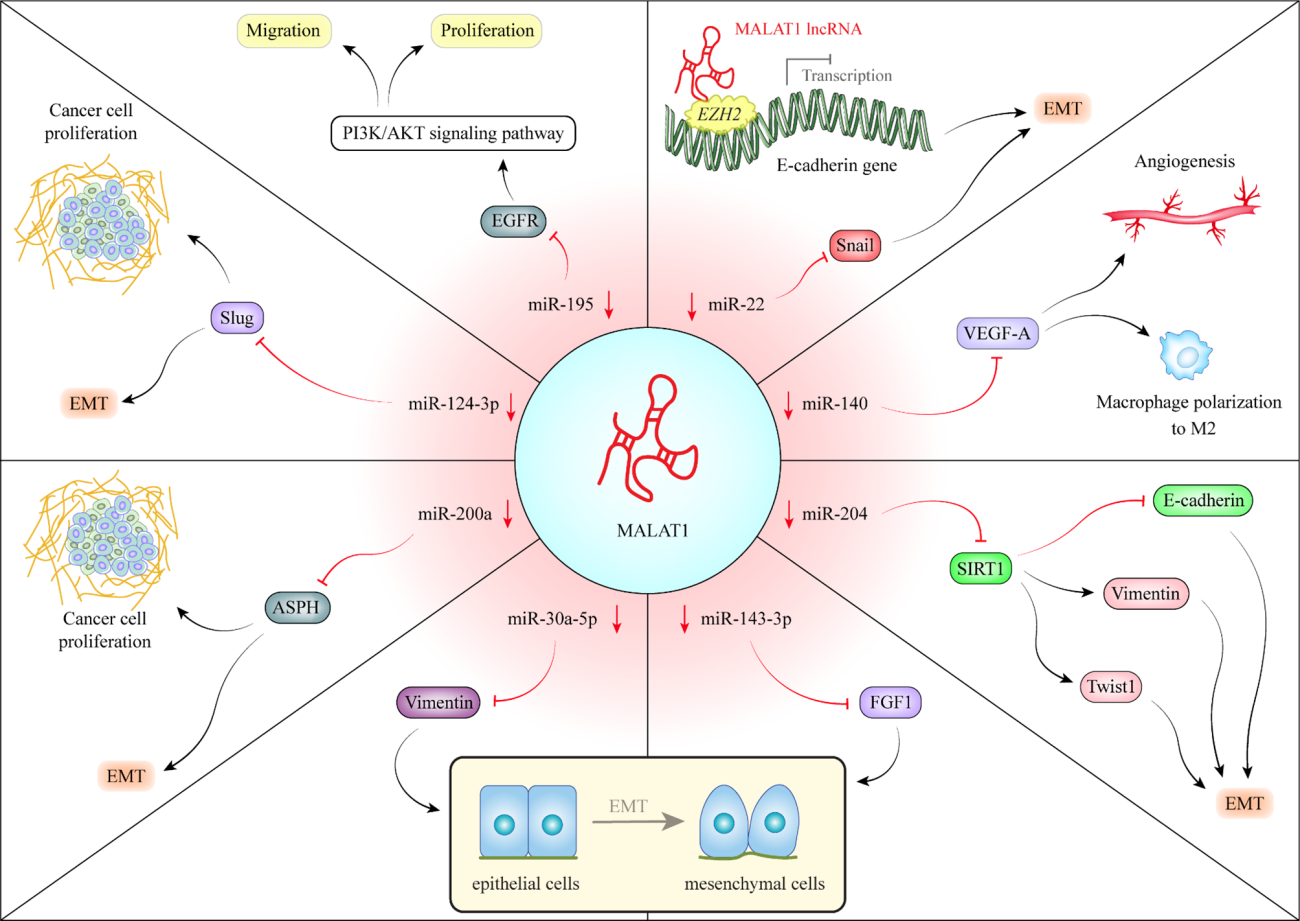
MALAT1 is an important oncogenic lncRNA in hepatocellular carcinoma (HCC). MALAT1 can sequester several miRNAs. For instance, MALAT1 can sequester miR-140. Through down-regulation of miR-140, MALAT1 enhances expression of VEGF-A and increases angiogenic potential. Moreover, *via* this route, MALAT1 enhances polarization of macrophage differentiation to M2. These macrophages facilitate tumor progression *via* modulation of tumor microenvironment (16). MALAT1 also reduces expression of miR-204 in HCC leading to upsurge in SIRT1 levels. SIRT1 up-regulation enhances expression of Vimentin and Twist and inhibits E-cadherin, thus facilitating epithelial-mesenchymal transition (EMT) (17). MALAT1 can also sequester miR-143-3p, thus up-regulating FGF1, N-cadherin, Vimentin, Snail, and Slug while down-regulating E-cadherin. These effects are associated with enhancement of EMT (18). Similarly, through down-regulation of miR-30a-5p, MALAT1 enhances Vimentin levels and EMT process (19). Via sequestering miR-200a, MALAT1 increases aspartate-β-hydroxylase (ASPH) levels, thus elevating expression of proteins which are involved in EMT or cell proliferation (20). MALAT1-mediated down-regulation of miR-124-3p leads to up-regulation of Slug, therefore increasing cell proliferation and EMT (21). MALAT1 can also sponge miR-195 resulting in over-expression of FGFR, activation of PI3K/AKT and enhancement of cell proliferation and invasion (22). Finally, MALAT1-mediated down-regulation of miR-22 increases Snail levels and facilitates EMT. Moreover, MALAT1 recruits EZH2 to the of promoter E-cadherin and miR-22 to decrease their expression (23). **Table 1** enlists function of over-activated lncRNAs in HCC.

The LINC01138 is located in a frequently amplified region in HCC. This lncRNA transcript is stabilized by IGF2BP1/IGF2BP3. Over-expression of LINC01138 in HCC confers malignant characteristics and is associated with poor survival of patients. Mechanistically, this lncRNA interacts with arginine methyltransferase 5 and increases the stability of this protein through inhibiting ubiquitin-mediated degradation in proteasomes (11). Over-expression of the lnc-Epidermal Growth Factor Receptor (EGFR) regulatory T cells (Tregs) has been related with tumor size and levels of EGFR/Foxp3. Its over-expression has also been negatively correlated with the levels of interferon (IFN)-γ in HCC patients and animal models. This lncRNA promotes Treg differentiation, inhibits function of cytotoxic T cells and increases HCC growth. These effects are exerted through binding of lnc-EGFR with EGFR, increasing its stability and activation of the AP-1/NF-AT1 axis (12). The oncogenic lncRNA HULC has been shown to exert its effects *via* modulation of phosphorylation pattern of YB-1. Notably, up-regulation of this lncRNA in HCC has been correlated with pathological grade and patients’ outcome. HULC can also increase cell proliferation, migration, and invasion and suppress cisplatin-associated cell apoptosis (13). LncRNA-MUF is another over-expressed lncRNA in HCC tissues whose up-regulation has been correlated with poor clinical outcome. This lncRNA has an indispensable impact in epithelial-mesenchymal transition (EMT). Such effects have been exerted through binding with Annexin A2 and induction of the Wnt/β-catenin signaling. Mechanistically, lncRNA-MUF serves as a competing endogenous RNA (ceRNA) for miR-34a, resulting in up-regulation of Snail1 induction of EMT process (14). GHET1 over-expression in HCC sections has been associated with vascular invasion, cirrhosis, size of tumor, histological grade, and poor clinical outcome. GHET1 silencing has suppressed cell proliferation and prompted both cell cycle arrest and cell apoptosis. GHET1 can suppress expression of KLF2 in HCC cells through recruitment of PRC2 into its promoter (15). MALAT1 is another up-regulated lncRNA in HCC, which affect neoplastic transformation through several mechanisms among them is its role as a ceRNA. **Figure 1** depicts this mechanism in HCC.

**Table 1:** Function of over-activated lncRNAs in HCC (ANT, adjacent non-cancerous tissue; HBS Ag, hepatitis B surface antigen).

lncRNA	Sample	Cell line	Interacting partners	Signaling pathway	Association with clinical features	Function	Reference
*NEAT1*	40 HCC tissues and paired ANTs, Male BALB/c nude mice	L02, 293 T, HepG2, Huh7, SK-Hep-1, HCCLM3	miR-124-3p, ATGL	–	Patient survival	Promotes HCC cell growth through miR-124-3p-mediated downregulation of ATGL.	(24)
*NEAT1*	30 HCC tissues and paired ANTs, BALB/c athymic nude mice	HepG2, L02, Huh7,	miR-129-5p, VCP, IκB	–	–	Enhances proliferation of HCC cells *via* affecting miR-129-5p-VCP-IκB.	(25)
*NEAT1*	–	Huh7, Hep3B, HepG2, Bel-7404, SK-Hep1, LO2, HEK-293T	miR-485, STAT3	–	–	Contributes to evolution of HCC through sequestering miR-485 and upregulation of STAT3.	(26)
*NEAT1*	86 HCC tissues and paired ANTs	SMMC-7721, Huh-7, Hep3B, THLE-2	–	–	Patient survival, liver cirrhosis, microvascular invasion, TNM stage	Promotes proliferation HCC cells	(27)
*NEAT1*	62 HCC tissues and paired ANTs	MHCC97H, MHCC97L, SMCC7721, Huh7, LO2	miR-613	–	tumor size, vascular invasion	Stimulates proliferation and invasion *via* regulating miR-613	(28)
*NEAT1*	12 female BALB/c, nude mice	Hep3B, LM3, MHCC97L, SK-hep1, HepG2, LO2, HEK-293T	hsa-miR-139-5p, TGF-β1	–	–	Promotes HCC progression *via* sequestering hsa-miR-139-5p and upregulation of TGF-β1	(29)
*NEAT1_2*	21 HCC tissues and paired ANTs	LO2, Huh7, SMMC-7721, PLC5, Bel-7402	miR-101-3p, WEE1	–	–	Reduces radiosensitivity through miR-101-3p- WEE1 axis	(30)
*PTTG3P*	46 HCC tissues and paired ANTs, 90 paraffin-embedded tissues and ANTs, male BALB/C nude mice	HepG2, Hep3B	PTTG1	PI3K/AKT signaling pathway	Patient survival, tumor size, TNM stage	Stimulates proliferation, migration and invasion and blocks apoptosis *via* upregulating PTTG1	(31)
*PTTG3P*	50 HCC tissues and paired ANTs, female nude mice	HepG2, Hep3B, Huh-7, HLF, SK-HeP-1, SNU-449, LO2	CCND1, PARP2, miR-383	PI3K/AKT	tumor size, tumor stage, metastasis	Promotes proliferation, migration, and invasion and inhibits apoptosis in HCC cells.	(32)
*UBE2CP3*	46 HCC tissues and ANTs, male BALB/c nude mice	HepG2, SMMC-7721, HUVEC	–	ERK/HIF-1α/p70S6K/VEGFA signaling	Patient survival, tumor invasion, tumor number	Promotes migration, invasion, and angiogenesis through activating ERK/HIF-1α/p70S6K/VEGFA signaling	(33)
*LINC00461*	87 HCC tissues and paired ANTs, mice	Huh7, SMMC-7721, MHCC97H, Hep3B, HepG2, LO2	miR-149-5p, LRIG2	–	Advanced stage, metastasis	Promotes proliferation, migration, and invasiveness *via* miR-149-5p- LRIG2 axis	(34)
*MALAT1*	20 HCC tissues and paired ANTs, female Nude mice	LO2 cells, HepG2 cells, Huh-7 cells, THP-1, HUVEC	miR-140, VEGF-A	–	–	Promotes angiogenesis of HCC cells through targeting miR-140 and surging VEGF-A	(16)
*MALAT1*	20 HCC tissues and paired ANTs	LO2, Bel7404, Huh7, HepG2	miR-204, SIRT1	–	–	Promotes migration and invasion of HCC cells through sponging miR-204 and surging SIRT1	(17)
*MALAT1*	56 HCC tissues and paired ANTs	Huh-6, HepG2, SMMC-7721, Bel-7402, LO2	miR-143-3p, ZEB1	–	Patient survival, TNM stage, distant metastasis	Promotes HCC development *via* sequestering miR-143-3p and regulation of ZEB1	(35)
*MALAT1*	30 HCC tissues and paired ANTs, male BALB/c nude mice	HepG2, Huh7, HEK-293T	miR-30a-5p, Vimentin	–	–	Promotes migration and invasion in HCC cells *via* affecting miR-30a-5p/Vimentin axis	(19)
*MALAT1*	–	Huh7, SNU-423, PLC, Hep3B	miR-200a	–	–	Regulates proliferation, migration, and invasion under hypoxic condition through sponging miR-200a.	(36)
*MALAT1*	15 HCC tissues and paired ANTs, male BALB/c nude mice	HepG2, HuH7, HEK293T	miR-124-3p, Slug	–	Patient survival, tumor size, MVI, differentiation status	Promotes migration and invasion of HCC cells through influencing miR-124-3p/Slug axis	(21)
*MALAT1*	–	L-02, QSG-7701, HepG2, MHCC97	miR-195, EGFR	PI3K/AKT and JAK/STAT signaling pathways	–	Promotes growth and motility of HCC cells through regulation of miR-195/EGFR axis	(22)
*MALAT1*	30 HCC tissues and paired ANTs, female BALB/c nude mice	HepG2, Hep3B, HuH7, PLC/PRF5	miR-22, SNAI1	–	–	Contributes to HCC progression though sponging miR-22 and upregulation of SNAI1 expression	(23)
*MNX1-AS1*	81 HCC tissues and paired ANTs, mice	Huh7, SMMC-7721, MHCC97H, Hep3B, HepG2, and LO2	miR-218-5p, COMMD8	–	Patient survival, TNM stage, metastasis	Enhances proliferation and invasion of HCC cells through targeting miR-218-5p and inhibition of COMMD8	(37)
*MCM3AP-AS1*	80 HCC tissues and paired ANTs, male BALB/c nude mice	LO2, HepG2, Hep3B, Huh7, SMMC-7721	miR-194-5p, FOXA1	–	Poor prognosis, tumor size, tumor grade, advanced tumor stage	Promotes proliferation, colony formation, and cell cycle transition and decreases apoptosis in HCC cells	(38)
*MCM3AP-AS1*	25 HCC tissues and paired ANTs	HepG2, Huh-7, 293T	miR-455	–	Patient survival	Promotes HCC metastasis through interacting with and regulation of EGFR expression	(39)
*TUG1*	77 HCC tissues and paired ANTs, male BALB/c nude mice	HepG2, MHCC-97H, Hep3B, L02	KLF2	–	tumor size, BCLC stage	Promotes HCC cells proliferation through epigenetically repression of KLF2	(40)
*TUG1*	HCC tissues and paired ANTs	–	miR-455-3p, AMPKβ2	–	Patient survival	Affects cell growth, metastasis, and glycolysis *via* miR-455-3p/AMPKβ2 axis	(41)
*TUG1*	92 HCC tissues and paired ANTs, female BALB/c athymic nude mice	HepG2, Hep3B, SMMC-7721, HCCLM3, Bel-7402	miR-142-3p, ZEB1	–	–	Contributes to metastasis and EMT process in HCC through downregulation of miR-142-3 and regulation of ZEB expression	(42)
*TUG1*	41 HCC tissues and paired ANTs, female BALB/C athymic nude mice	Hep3B, Huh7, Bel7402, HepG2, SMMC-7721, HL7702	miR-144	JAK2/STAT3 signaling pathway	–	Promotes proliferation, migration, and tumorigenesis *via* interacting with miR-144	(43)
*THOR*	80 HCC tissues and paired ANTs, nude mice	HCCLM3, SMMC7721	–	PTEN/AKT signaling pathway	Patient survival	Enhances proliferation and metastasis of HCC cells by amplifying PTEN/AKT signaling	(44)
*ANRIL*	FFPE specimens of 43 pairs of HCC tissues and ANTs, male athymic BALB/c nude mice	Huh7, SMMC7721, HepG2, Hep3B, L02	miR-199a-5p, ARL2	–	–	Enhances mitochondrial function in HCC cells through regulation of miR-199a-5p/ARL2 axis	(45)
*ANRIL*	–	HepG2	miR-191	–	–	Promotes proliferation, migration, and invasion and reduces apoptosis in HCC cells through downregulation of miR-191	(46)
*ANRIL*	77 HCC tissues and paired ANTs, male BALB/c nude mice	HepG2, Hep3B, MHCC-97H	KLF2	–	tumor size, BCLC stage	Promotes proliferation, invasion, and reduces apoptosis in HCC cells	(47)
*ANRIL*	31 HCC tissues and paired ANTs, female BALB/C athymic nude mice	SMMC772, HUH7, Hep3B, HepG2	miR-122-5p	–	–	Promotes proliferation, metastasis and invasion of HCC cells *via* affecting miR-122-5p expression	(48)
*ANRIL*	130 tissues and paired ANTs	HepG2	–	–	Patient survival, histologic grade, TNM stage	Promotes proliferation, migration, and invasion of HCC cell.	(49)
*ANRIL*	–	MHCC97, Li-7, THLE-3	miR-144, PBX3	PI3K/AKT and JAK/STAT signaling pathways	–	Surges proliferation, migration, and invasion of HCC cells through sponging miR-144 and upregulation of PBX3	(50)
*AWPPH*	88 HCC tissues and paired ANT, male athymic BALB/c nude mice	QSG-7701, SMMC-7721, HCCLM3, Huh7, HepG2	YBX1, SNAIL1, PIK3CA	PI3K/AKT signaling pathway	Patient survival, encapsulation incomplete, microvascular invasion, TNM stage, BCLC stage	Promotes proliferation and migration of HCC cells through YBX1-mediated activation of SNAIL1 translation and PIK3CA transcription	(51)
*PVT1*	47 HCC tissues and paired ANTs, nude BALB/c male mice	L-02, SK-HEP-1, Hep G2, SMMC-7721, BEL-7402, Hep3B2.1-7, QGY-7703X4	miR-150, HIG2	–	–	Promotes proliferation, migration and invasion, and induced cell apoptosis in HCC cells through regulation of miR-150/HIG2 axis	(52)
*PVT1*	48 HCC tissues and paired ANTs	HepG2, Hep3B, Huh-7, HCCLM9, SK-Hep1, SMMC-7721	miR-186-5p, YAP1	–	Patient survival, vascular invasion, liver cirrhosis, TNM stage	Promotes proliferation, migration, and invasion through targeting miR-186-5p and enhancement of YAP1	(53)
*PVT1*	80 HCC tissues and paired ANTs	Bel-7402, Huh7, Hep3B, HepG2	miR-365, ATG3		TNM stage, tumor size	Promotes autophagy in HCC cells *via* sponging miR-365 and upregulation of ATG3	(54)
*SNHG1*	Male BALB/c nude mice	HL-7702, s Li-7, HuH7, HHCC, H-97, Hep3b, SMMC-7721	miR-195-5p, PDCD4	–	–	Promotes proliferation and migration of HCC cells through targeting miR-195-5p and upregulation of PDCD4	(55)
*SNHG1*	82 HCC tissues and paired ANTs	SMMC-7721, MHCC97H, HCCLM3, HepG2, QSG-7701, L02	p53	–	Patient survival, tumor size, tumor differentiation, BCLC stage	Stimulates proliferation, cell cycle progression, and blocks apoptosis in HCC cells *via* inhibiting p53	(56)
*SNHG1*	122 HCC tissues and paired ANTs	HepG2	miR-195	–	tumor size, TNM stage	Stimulates proliferation, migration, and invasiveness of HCC cells through inhibiting miR-195	(57)
*ENST00000429227.1*	161 HCC tissues and paired ANTs	U937	–	–	Patient survival, surgical margin, AFP, BCLC stage	Is associated with poor prognosis in HCC	(58)
*H19*	42 HCC tissues and paired ANTs	Huh 7	–	MAPK/ERK signaling pathway	–	Its downregulation induces oxidative stress and reduces chemotherapy resistance of HCC cells.	(59)
*H19*	46 HCC tissues and paired ANTs	linesHepG2, SMMC-7721, Bel-7402, Huh-7	miR-15b, CDC42	CDC42/PAK1 pathway	–	Promotes proliferation, migration, and invasion and reduces apoptosis in HCC cells through regulating miR-15b/CDC42 axis.	(60)
*H19*	–	HepG2, MHCC-97L, SK-hep1, Hun7, SMCC-7721, LO2, HEK- 293T	miR-326, TWIST1	–	–	Promotes proliferation, migration, and invasion of HCC cells through regulating miR-326/TWIST1 axis.	(61)
*HCG11*	20 HCC tissues and paired ANTs	L-02, Huh7, HepG2, SMMC-7721, SK-HEP-1	IGF2BP1	MAPK signaling pathway	–	Promotes proliferation, migration, and invasion and reduces apoptosis in HCC cells	(62)
*LINC00665*	76 HCC tissues and paired ANTs, 24 female BALB/c nude mice	Huh-7, HepG2, HCCLM6, MHCC-97H, Hep3B, HL-7702	miR-186-5p, MAP4K3	–	Patient survival, tumor size, Edmondson grade	Enhances cell viability and decreases apoptosis and autophagy through regulation of miR-186-5p/MAP4K3 axis	(63)
*CRNDE*	46 HCC tissues and paired ANTs	HepG2, Huh-7, HCCLM3, SNU449, SNU475, HepaRG, HL‐7702	miR-217, MAPK1	–	AJCC stage, vascular invasion, distant metastasis	Promotes proliferation, migration and invasion in HCC cells *via* affecting miR-217/MAPK1 axis	(64)
*CRNDE*	23 HCC tissues and paired ANTs, BALB/c (nu/nu) mice	QSG-7701, HepG2, Hep3B, Huh7	–	PI3K/Akt and Wnt/β-catenin signaling pathways	Patient survival	Promotes proliferation of HCC cells through regulation of mentioned signaling pathways	(65)
*CRNDE*	12 HCC tissues and paired ANTs, male BALB/c nude mice	SMMC7721, SK-hep1, Huh7, HepG2	miR-136-5P, IRX5			Affects proliferation, migration, and invasiveness of HCC cells *via* targeting miR-136-5P and regulation of IRX5	(66)
*CRNDE*	25 HCC tissues and paired ANTs, 10 female athymic BALB/c nude mice	HepG2, Huh7, L-02	miR-203, BCAT1	–	–	Affects proliferation, migration, and invasiveness of HCC cells by regulating miR-203/BCAT1 axis	(67)
*CRNDE*	60 HCC tissues and paired ANTs, male athymic BALB/c nude mice	HL7702, MHCC97H, HCCLM6, SNU-398, Huh7	miR-337-3p, SIX1	–	–	Promotes HCC progression through sponging miR-337-3p and upregulation of SIX1	(68)
*FOXD2-AS1*	18 HCC tissues and paired ANTs	L-02, HepG2, Huh-7, SMMC-7721, Bel-7402, Hep3B	miR-185, AKT	–	–	Supports proliferation and metastasis of HCC cells through regulation of miR-185/AKT axis	(69)
*FOXD2-AS1*	88 HCC tissues and paired ANTs	L-02, HepG2, Hep3B, SMMC-7721, LM3	DKK1	Wnt/β-catenin signaling pathway	Patient survival	Contributes to progression of HCC by epigenetically silencing DKK1 and activation of Wnt/β-catenin signaling pathway	(70)
*LINC00488*	46 HCC tissues and paired ANTs, 30 nude mice	L02, Huh-7, Hep3B, HCCLM3, MHCC97	miR-330-5p, TLN1	–	–	Promotes proliferation and angiogenesis of HCC cells through miR-330-5p-mediated upregulation of TLN1	(71)
*AY927503*	57 HCC tissues and paired ANTs, female BALB/c nude mice	Hep3B, HepG2, SK-Hep1, LM3, BEL-7404, SMMC-7721, LO2, HUVEC, HEK-293T	ITGAV	–	Patient survival	Enhances cell migration, drug resistance, and metastasis in HCC cells through activation of ITGAV transcription	(72)
*lncRNA-PE*	24 HCC tissues and paired ANTs	BEL-7402, SK-Hep-1, LO2	miR-200a/b, ZEB1	–	–	Enhances migration, invasion and EMT process in HCC cells through miR-200a/b/ZEB1 axis	(73)
*HULC*	30 HCC tissues and paired ANTs	HepG2, SMMC7721, LO2	miR-372-3p, Rab11a	–	TNM stage	Promotes proliferation and invasion and suppresses apoptosis through sponging miR-372-3p and upregulation of Rab11a	(74)
*HULC*	male athymic Balb/C mice	Hep3B	miR-15a, P62, PTEN	AKT-PI3K-mTOR signaling pathway	–	Contributes to HCC progression through regulation of miR-15a, P62 and PTEN	(75)
*HULC*	41 HCC tissues and paired ANTs	HepG2, SMMC-7721	YB-1	–	Patient survival, differentiation	Promotes proliferation, migration and invasion and suppresses cisplatin-induced apoptosis in HCC cells	(76)
*SBF2-AS1*	18 male Balb/c nude mice	HepG2, Hep3B, SUN475, BEL-7405, BEL7404, BEL-7402, THLE-3	miR-140-5p, TGFBR1	–	Patient survival, lymph node metastasis, histologic grade, TNM stage	Contributes to evolution of HCC *via* sponging miR-140-5p and upregulation of TGFBR1	(77)
*SBF2-AS1*	134 HCC tissues and paired ANTs	HCCLM3, Huh7, SK-Hep1, HepG2, L02	–	–	vein invasion, TNM stage	Affects proliferation, migration and invasion of HCC cells	(78)
*UC001kfo*	82 HCC tissues and 20 ANTs, SPF BALB/c nude mice	GSG701, Hep3B, HepG2, Huh7, SMMC 7721, HCC LM3, L02	α-SMA	–	Patient survival, macro-vascular invasion, TNM stage	Affects proliferation, metastasis and EMT process through targeting α-SMA	(79)
*HOTTIP*	20 HCC tissues and 20 ANTs, male BALB/C nude mice	BEL7402, MHCC97H	miR-125b, HOXA	–	–	Promotes proliferation, migration, and tumorigenesis of HCC cells.	(80)
*FOXD2-AS1*	140 HCC tissues and paired ANTs, 12 female BALB/c nude mice	Hep3B, MHCC97-L, MHCC97-H, SK-HEP1, HCCLM3, HL7702	miR−206, ANXA2	–	Patient survival	Increases cell viability and metastasis of HCC cells through miR−206/ANXA2 axis	(81)
*LUCAT1*	90 HCC tissues and paired ANTs, male BALB/c nude mice	HepG2, SMMC-7721, SNU‐423, Hep3B, Huh7, MHCC‐97H, L02	–	–	Patient survival, tumor size, metastasis, TNM stage	Affects proliferation and metastasis of HCC cells through inhibition of phosphorylation of ANXA2	(82)
*AK001796*	73 HCC tissues and paired ANTs	LO2, g SMMC-7721, Huh-7, MHCC-97H, MHCC-97L	–	–	Patient survival, tumor size, TNM stage	Promotes proliferation and invasion of HCC cells	(83)
*FEZF1-AS1*	139 HCC tissues and paired ANTs, male BALB/c nude mice	HepG2, SK-hep1, Huh7, HCCLM3, LO2	–	JAK2/STAT3 signaling pathway	Patient survival, tumor size, TNM stage, venous invasion	Promotes proliferation, migration and invasion of HCC cells	(84)
*MINCR*	161 HCC tissues and paired ANTs	–	–	–	Patient survival, TNM stage, histological grade	Contributes to progressive clinicopathological features and poor survival	(85)
*MINCR*	70 primary HCC tissues and paired ANTs	SMMC-7721, Huh7, HCC-LM3, HepG2, MHCC-97H, HL-7702	–	–	–	Promotes proliferation, migration, and invasion.	(86)
*LINC01152*	30 pairs of HBV-HCC related tissues and HCC tissues, nude mice	Huh7, HepG2, Hep3B	IL-23	–	–	Enhances proliferation, survival and tumor formation ability through IL-23	(87)
*XIST*	88 HCC tissues and paired ANTs, male BALB/c-nu/nu mice	LM9, Hh7, Hep3B, HepG2, LO2	miR-139-5p, PDK1	AKT signaling pathway	Patient survival, tumor size	Promotes cell proliferation and reduces apoptosis through regulation of miR-139-5p/PDK1/AKT axis	(88)
*XIST*	52 HCC tissues and paired ANTs	MHCC97L, MHCC97H, HepG2, SMMC7221, Huh7, Bel-7402, HL-7702	miR-194-5p, MAPK1	–	Patient survival, tumor size, vascular invasion	Promotes proliferation, migration and invasion of HCC cells through sponging miR-194-5p and regulation of MAPK1 expression	(89)
*TRPM2-AS*	108 HCC tissues and paired ANTs	HCCLM3, Huh7, SMMC-7721, SKHep1, HepG2, QSG7701			Patient survival, tumor size, AJCC stage, tumor differentiation	Promotes proliferation and reduces apoptosis in HCC cells	(90)
*LINC-ITGB1*	56 HCC tissues and paired ANTs, blood samples from 80 HCC patients and 44 healthy controls	C3A, HEP G2, m, THLE-3	ROCK1	–	–	Promotes proliferation, migration and invasion of HCC cells through upregulation of ROCK1	(91)
*LSINCT5*	126 HCC tissues and paired ANTs, female nude mice	97L, HepG2, Hep3B, 7721, and Huh7, 293T, L02	miR-4516, HMGA2	–	Patient survival, TNM stage, tumor size, metastasis	Promotes HCC progression through regulation of miR-4516/HMGA2 axis	(92)
*XLOC*	68 HCC tissues and paired ANTs	HepG2, Hep3B, SMMC-7721, Bel-7402	–	–	Patient survival, vascular invasion tumor size, Edmindson grade	Promotes proliferation and migration and reduces apoptosis in HCC cells	(93)
*HNF1A-AS1*	–	SMMC-7721, Huh7, MHCC97L, HepG2, LO2	NKD1, p21	–	–	Promotes proliferation of HCC cells through inhibition of NKD1 and p21 *via* interacting with EZH2	(94)
*HNF1A-AS1*	40 HCC tissues and paired ANTs	HepG2, SMMC-7721, PLC/PRF/5, Huh7, HL7702	hsa-miR-30b-5p, ATG5	–	tumor size, multiplicity of tumors, histological differentiation, TNM stage	Reduces apoptosis rate and promotes autophagy in HCC cells through sponging hsa-miR-30b-5p	(95)
*URHC*	52 HCC tissues and paired ANTs	HepG2, SMMC7721, Huh7, HL-7702	ZAK	ERK/MAPK signaling pathway	Patient survival, tumor size, tumor number	Promotes cell proliferation and inhibits apoptosis through suppression of ZAK	(96)
*UCA1*	60 HCC tissues and paired ANTs, male BALB/C nude mice	LO2, MHCC97L, Huh7, MHCC97H, SK-hep1	miR-203, Snail2	–	tumor size, vascular invasion, AJCC stage	Promotes HCC progression through targeting miR-203 and upregulation of Snail2	(97)
*AK021443*	20 HCC tissues and paired ANTs, male BALB/c-nu/nu mice	Bel-7402, Sk-Hep1, HepG2, Huh-7, Bel-7404, LO2	–	–	–	Promotes proliferation, migration, invasion and colony formation in HCC cells	(98)
*RUSC1-AS-N*	66 HCC tissues and paired ANTs	QSG-7701, SMMC-7721, HCCLM3, Huh7	–	–	Patient survival, tumor size, vein invasion, encapsulation, BCLC stage	Promotes cell viability and reduces apoptosis and cell cycle arrest	(99)
*CCAT1*	40 HCC tissues and paired ANTs	MHCC97H, MHCC97L, Hep3B, SMCC-7721, LO2	miR-490-3p, CDK1	–	tumor site, AJCC stage	Promotes proliferation and invasion of HCC cells through targeting miR-490-3p and regulation of CDK1	(100)
*CCAT1*	66 HCC tissues and paired ANTs	LO2 and QSG-7701, SMMC-7721, Hep3B, Huh7, HepG2	let-7, HMGA2, c-Myc	–	Patient survival, tumor size, microvascular invasion, AFP	Enhances proliferation and migration of HCC cells through sponging let-7 and regulation of HMGA2 and c-Myc expression	(101)
*CCAT1*	39 HCC tissues and paired ANTs	HCCLM3, Huh7, Hep3B, HepG2, L02	miR-181a-5p, ATG7	–	–	Promotes autophagy and proliferation in HCC cells through sponging miR-181a-5p and regulation of ATG7 expression	(102)
*CCAT1*	65 HCC tissues and 35 normal liver samples	Hep3B	miR-30c-2-3p, CCNE1	–	metastasis	Promotes HCC cells proliferation by sequestering miR-30c-2-3p and upregulation of CCNE1	(103)
*CCAT2*	20 HCC tissues and paired ANTs, male BALB/c-nude mice	SMMC7721, SK-hep1, HepG2, Huh7, L02	NDRG1	–		Stimulates proliferation and metastasis of HCC cells through upregulation of NDRG1	(104)
*SNHG16*	71 HCC tissues and paired ANTs	HL-7702, SK-Hep-1, Huh7, Hep3B, HepG2	–	–	Patient survival, tumor size, AFP level, PVTT, metastasis	Promotes proliferation, migration and invasion and increases sorafenib resistance in HCC cells	(105)
*SNHG16*	40 HCC tissues and paired ANTs, BALB/c nude mice	HepG2, SMMC7721, Hep3B, Bel7402, Huh7, LO2	miR-195	–	TNM stage, metastasis	Enhances proliferation, invasion and tumorigenesis of HCC cells through targeting miR-195	(106)
*SNHG10*	64 HCC tissues and paired ANTs	SNU-182, Huh-7, Hep3B, SK-Hep1, and SNU-387, HEK293T, HCCLM3	miR-150-5p, SCARNA13	–	Patient survival	Contributes to HCC progression and metastasis through modulating SCARNA13	(107)
*SNHG12*	48 HCC tissues and paired ANTs	SK-Hep1	miR-199a/b-5p, MLK3	NF-κB signaling pathway	Patient survival, tumor size, vascular invasion, TNM stage	Enhances tumorigenesis and metastasis of HCC cells *via* targeting miR-199a/b-5p	(108)
*SNHG20*	96 HCC tissues and paired ANTs	LO2, MHCC97L, SMCC7721, MHCC97H, Huh-7	EZH2, E-cadhein	–	Patient survival, tumor size, TNM stage	Promotes proliferation and invasion of HCC cells through binding to EZH2 and regulation of E-cadherin expression	(109)
*SNHG5*	48 HCC tissues and paired ANTs	Hep3B, HepG2, SMCC-7721, MHCC-97L, MHCC-97H, Huh7, LO2	miR-26a-5p, GSK3β	Wnt/β-catenin signaling pathway	Patient survival, tumor size, HBV infection, histologic grade, TNM stage	Promotes HCC progression and metastasis through targeting miR-26a-5p and regulation of GSK3β	(110)
*SNHG6*	Expression data of HCC obtained from TCGA and GEO	MHCC-97H, HCC-LM3	let-7c-5p, c-Myc	–	Patient survival	Enhances proliferation of HCC cells through sponging let-7c-5p and upregulation of c-Myc	(111)
*SNHG6*	12 HCC tissues and paired ANTs, female BALB/c mice	HL-7702, HepG2, Hep3b, HLE, Huh-7	miR-139-5p, SERPINH1	–	–	Promotes HCC progression *via* targeting miR-139-5p and regulation of SERPINH1	(112)
*SNHG6-003*	52 HCC tissues and paired ANTs, FFPE tissues from 160 patients	BEL-7402, SMMC-7721, MHCC-97H, SK-Hep-1, Huh7, HCC-LM3	miR-26a/b, TAK1	–	Patient survival, portal vein tumor thrombus, Barcelona Clinic Liver Cancer stage, distant metastasis	Promotes HCC cells proliferation and drug resistance by sponging miR-26a/b and upregulation of TAK1	(113)
*SNHG7*	40 HCC tissues and paired ANTs, male BALB/c nude mice	HepG2, HCC-LM3	miR-425	Wnt/β-catenin/EMT signaling pathway	Patient survival	Enhances proliferation, migration and invasiveness *via* sponging miR-425 and regulation of Wnt/β-catenin/EMT signaling pathway	(114)
*SNHG7*	80 HCC tissues and paired ANTs, BALB/C nude mice	LO2, Hhu7, Hep3B, HCCLM3, MHCC97H	miR-122-5p, RPL4	–	Patient survival, tumor stages, tumor grades, vascular invasion	Promotes proliferation, migration and invasiveness *via* affecting miR-122-5p and RPL4	(115)
*SNHG8*	23 HCC tissues and paired ANTs, female immune-deficient nude mice	LO2, Huh6, Huh7, SK-hep1, HepG2, PLC5	miR-149	–	Recurrence	Promotes Tumorigenesis and metastasis through sponging miR-149	(116)
*SNHG15*	101 HCC tissues and paired ANTs	HuH-1, HuH-7, L-O2	miR-490-3p, HDAC2	–	Tumor size, Edmondson-Steiner grading, TNM stage	Promotes proliferation, migration and invasion *via* regulating miR-490-3p/HDAC2 axis	(117)
*CCAL*	37 HCC tissues and ANTs, 60 male nude mice	Huh7, HCCLM3, LO2	AP-2α	Wnt/β-catenin signaling pathway	tumor metastasis, TNM stage	Promotes proliferation and invasion of HCC cells through upregulation of AP-2α	(118)
*Sox2ot*	84 HCC tissues and ANTs	HepG2, SMMC-7721	–	–	Patient survival, histological grade, TNM stage, vein invasion	Promotes HCC cells metastasis	(119)
*SPRY4-IT1*	male nude mice	MHCC97H, MHCC97L, SKhep-1, LO2	E-cadherin	–	–	Stimulates proliferation and invasion of HCC cells *via* interaction with EZH2 and repression of E-cadherin levels	(120)
*SPRY4-IT1*	82 HCC tissues and paired ANTs	HL7702, MHCC97L, MHCC97H, HepG2, SMMC7721	ERRα	–	Patient survival, TNM stage, metastasis	Promotes proliferation, migration and invasion and decreases apoptosis *via* suppressing ERRα expression	(121)
*PANDAR*	482 HCC tissues and paired ANTs	HCCLM3, Hep3B, HepG2, Huh-7, MHCC97H, PLC, SMMC-7402, SMMC-7721	–	–	Patient survival, liver cirrhosis, HBs Ag, AFP, tumor nodule, vascular invasion, TNM stage	Promotes HCC tumorigenesis and is associated with poor prognosis	(122)
*linc-ROR*	female BALB/c nude mice	HepG2, SMMC-7721	miR-145, RAD18	–	–	Promotes metastasis, EMT process and radioresistant in HCC cells through targeting miR-145 and regulation of RAD18 expression	(123)
*CARLo-5*	97 HCC tissues and paired ANTs	HepG2, Hep3B, SK-HEP1, SMMC7721, MHCC97-L, MHCC97-H, PLC/PRF/5, HCCLM3	–	–	Patient survival, liver cirrhosis, tumor number, vascular invasion, capsular formation, Edmondson-Steiner grade	Promotes proliferation, migration and invasion of HCC cells	(124)
*AB019562*	50 HCC tissues and paired ANTs	SMMC-7721, PLC/PRF/5, C3AHCC, THLE-3, HepG2	–	–	–	Promotes proliferation, migration and invasive features and reduces apoptosis in HCC cells	(125)
*PlncRNA-1*	84 HCC tissues and paired ANTs, male BALB/c nu/nu mice	HCCLM3, Huh7, SK-Hep1, HepG2, L02	–	–	Patient survival, tumor size, vascular invasion, TNM stage	Promotes metastasis and EMT process in HCC cells and is correlated with poor prognosis	(126)
*lncRNA-TPTE2P1*	72 HCC tissues and 66 normal tissues,	HepG2, Huh7, MHCC97, Bel7402, SMMC7721, HCCLM3	–	–	tumor size, distant metastasis, differentiation degree, TNM stage	Promotes proliferation, migration and EMT process of HCC cells	(127)
*PCAT-1*	82 HCC tissues and paired ANTs	HepG2, Bel-7402	–	–	–	Increases proliferation and migration and inhibits apoptosis in HCC cells	(128)
*PCAT-14*	39 HCC tissues and paired ANTs	Huh7, HCCLM3, HepG2, SMMC7721, PLC5, QGY7701, LO2	miR-372	–	Patient survival, TNM stage, tumor metastasis, tumor size	Promotes proliferation and invasion of HCC cells through inducing methylation of miR-372	(129)
*BLACAT1*	37 HCC tissues and paired ANTs, male athymic nude (nu/nu) mice	HeG2, MHCC97L, HuH7, Hep3B, SK-HEP-1, SNU-449, SNU-182, SNU-429, bel-7402, THLE2, THLE3	has-miR-485-5p	–	–	Promotes proliferation and invasion in HCC cells *via* upregulation of has-miR-485-5p.	(130)
*DLX6-AS1*	60 HCC tissues and paired ANTs, 20 male BALB/c nude mice	MHCC97L, HCCLM3, HepG2, Hep3B, Huh7, LO2	miR-203a, MMP-2	–	tumor size, Edmondson grading, TNM stage	Contributes to HCC progression *via* regulating miR-203a/MMP-2 axis	(131)
*RAB5IF*	–	HepG2, Hep3B, Huh7, MCF-7, A549, HeLa	LGR5	–	–	Promotes HCC progression *via* LGR5 mediated elevation of β-catenin and c-Myc	(132)
*LOC90784*	64 HCC tissues and paired ANTs	L02, HepG2, SMMC7721, Bel-7404, PLC/PRF/5	–	–	Patient survival, tumor differentiation, TNM stage, venous invasion, HBV status, serum AFP	Promotes cell proliferation, migration and invasion and reduces apoptosis	(133)
*HOTAIR*	53 HCC tissues and paired ANTs	HepG2, Bel-7402	RBM38	–	–	Enhances migration and invasion of HCC cells *via* regulating RBM38	(134)
*HOTAIR*	30 HCC tissues and paired ANTs, female BALB/c nude mice	HepG2, Huh7, Hep3B, SMMC7721, MHCC97H, MIHA	miR-122	–	–	Promotes cell proliferation and reduces cell cycle arrest through upregulation of miR-122	(135)
*BZRAP1-AS1*	49 HCC tissues and paired ANTs, 90 specific pathogen-free female nude mice	L-02, HuH-7, HCCLM3, LI7, BEL-7405, SK-HEP-1, BCLC-9	THBS1	–	tumor size, microvascular invasion, TNM stage	Promotes proliferation, migration and angiogenesis HCC cells through regulation of THBS1	(136)
*SNAI3-AS1*	46 HCC tissues and paired ANTs	MHCC‐97L, MHCC‐97h, HepG2, Hep3B, Huh7, L02	UPF1, Smad7	TGF-β/Smad signaling pathway	Patient survival, tumor size, TNM stage	Promotes proliferation, metastasis and EMT process *via* regulation of UPF1	(137)
*TP73-AS1*	84 HCC tissues and paired ANTs	HCCLM3, MHCC97L, SMMC7722, Hep3B,HepG2, THLE-3	miR-200a, HMGB1, RAGE	–	Patient survival, tumor size, tumor nodule number, TNM stage	Promotes proliferation of HCC cells through regulation of miR-200a/HMGB1/RAGE axis	(138)
*TP73-AS1*	72 HCC tissues and paired ANTs, male BALB/c nude mice	HL-7702, human HCC cell line HepG2, Hep3B, SMCC-7721	–	PTEN/Akt signaling pathway	–	Promotes cell proliferation and reduces apoptosis and radiosensitivity of HCC cells	(139)
*HANR*	35 HCC tissues and paired ANTs, male nude mice	Hep3B, Huh-7, LO-2	GSKIP, GSK3β	–	TNM stage, distant metastasis	Promotes cell growth, inhibits apoptosis and induces chemoresistance HCC	(140)
*MIAT*	45 HCC tissues and paired ANTs, BALB/c nude mice	HepG2, Huh7, SK-HEP-1, HLE, L02	miR-214	–	–	Promotes proliferation and invasion of HCC cells through sequestering miR-214	(141)
*MIAT*	20 HCC tissues and paired ANTs	HepG2, SMMC-7721, PLC/PRF/5, Huh7, SK-hep-1, 293T	miR-22-3p, sirt1	p53/p21 and p16/pRb signaling pathways	–	Its knockdown promotes cellular senescence and represses HCC tumorigenesis by regulating miR-22-3p/sirt1 axis	(142)
*lncRNA FAL1*	30 HCC tissues and paired ANTs	LO2, SMMC-7721, Huh7, HepG2, HepG2.2.15	miR-1236	–	Patient survival	Promotes proliferation and metastasis in HCC cells through targeting miR-1236	(143)
*CDKN2B-AS1*	100 HCC tissues and paired ANTs, 24 BALB/c male nude mice	LO2, HepG2, Huh7, SMMC-7721	let-7c-5p, NAP1L1	PI3K/AKT/mTOR signaling pathway	Patient survival, tumor size, microvascular invasion, tumor grade, tumor stage	Promotes tumor growth and metastasis of HCC through targeting let-7c-5p and upregulation of NAP1L1	(144)
*CDKN2B-AS1*	48 HCC tissues and paired ANTs	QGY-7703, PLC/ PRF/5, HB611, MHCC97	–	–	Patient survival, tumor size, TNM stage	Promotes HCC cells proliferation and is associated with poor prognosis	(145)
*CDKN2BAS*	85 HCC tissues and paired ANTs, nude mice	HCCLM3, SK-Hep-1, HUH7, MHCC97H, L02	miR-153-5p, ARHGAP18	MEK-ERK1/2 signaling pathway	–	Enhances proliferation and metastasis of HCC cells through sponging miR-153-5p and upregulation of ARHGAP18	(146)
*lncRNA-PDPK2P*	60 HCC tissues and paired ANTs, nude mice	MHCC97L, MHCC97H, BEL-7404, HCCLM3, SMMC7721	PDK1	PDK1/AKT/caspase 3 signaling pathway	Patient survival, tumor embolus, tumor differentiation	Promotes HCC progression through interaction with	(147)
*lncRNA Ftx*	73 HCC tissues and paired ANTs	LO2, Huh7, SMMC-7721, Bel-7402	–	–	–	Promotes proliferation, migration and invasion in HCC cells through PPARγ pathway	(148)
*MIR4435-2HG*	64 HCC tissues and paired ANTs	SNU-398, SNU-182	miRNA-487a	–	tumor size	Promotes proliferation of HCC cells through upregulation of miRNA-487	(149)
*SOX9-AS1*	67 HCC tissues and paired ANTs, male BALB/C nude	Huh7, HepG2, HCCLM3, Hep3B, L02	miR-5590-3p, SOX9	Wnt/β-catenin	Patient survival	Contributes to tumor growth and metastasis through sponging miR-5590-3p and upregulation of SOX9	(150)
*SOX21-AS1*	68 HCC tissues and paired ANTs	Hep3B, LM3, MHHC97H, HepG2, Huh7, LO2	p21	–	Patient survival, tumor size, Edminson Grade, vascular invasion, cirrhosis	Contributes to HCC progression through epigenetically silencing p21 by recruiting EZH2 to the promoter of p21	(151)
*HOXA11-AS*	66 HCC tissues and paired ANTs	HL-7702, HepG2, Hep3B, MHCC-97H, BEL7402	miR-124	–	Patient survival, tumor size, differentiation, TNM stage, lymph node metastasis, recurrence	Enhances migration and invasion of HCC cells through suppression of miR-124 by binding to EZH2	(152)
*HOXA-AS2*	58 HCC tissues and paired ANTs, female BALB/c nude mice	MHCC97L, Huh7, HepG2, HCCLM3, SMMC-7721, MHCC97H, HL-7702	miR-520c-3p, GPC3	–	–	Promotes migration and invasion of HCC cells through sponging miR-520c-3p and upregulation of GPC3	(153)
*HOXB-AS3*	36 HCC tissues and paired ANTs	HepG, PLC, Hep3B, LM3	p53	–	–	Its downregulation inhibits proliferation and induced apoptosis and cell cycle arrest in HCC cells through regulation of p53	(154)
*LINC00978*	33 HCC tissues and paired ANTs, sera of 58 HCC patients, 49 liver benign disease patients and 45 healthy controls, 10 BALB/c nude mice	7721, 7402, HepG2, LM3	EZH2, p21, E-cadherin	–	–	Promotes proliferation, migration, and invasion through epigenetically silencing of p21 and E-cadherin	(155)
*lncRNA-ATB*	72 HCC tissues and paired ANTs	SMMC-7721, HepG2	YAP, ATG5	–	Patient survival, tumor size, TNM stage	Promotes proliferation and clonogenicnity and also promotes autophagy by activating YAP and increasing ATG5 expression	(156)
*NR2F1-AS1*	47 HCC tissues from oxaliplatin-resistant and oxaliplatin‐sensitive, male nude mice	Huh7, HepG2, Lo-2	miR-363, ABCC1	–	–	Its knockdown suppresses migration, invasion and drug-resistant of HCC cells *via* regulating miR-363/ABCC1 axis	(157)
*DANCR*	Male athymic BALB/C nude mice	LO2, MHCC-97H, Huh7, HCC‐LM3, HepG2, MHCC‐97L, Hep3B, SMMC‐7721	miR-27a-3p	ROCK1/LIMK1/COFILIN1 pathway	Patient survival,	Enhances proliferation and metastasis and regulates EMT process through targeting miR-27a-3p	(158)
*DANCR*	BALB/c mice	Hep3B, HepG2, Huh7, SNU449, SK‐hep‐1, LO2	miR-216a-5p, KLF12	–	–	Promotes HCC malignancy and progression through sponging miR-216a-5p and regulation of KLF12 expression	(159)
*LINC00205*	80 HCC tissues and paired ANTs	LO2, Hep3B, Huh7, HEK293T	miR-122-5p	–	Tumor size, venous infiltration, TNM stage	Enhances proliferation, migration and invasion in HCC cells *via* miR-122-5p	(160)
*OSER1-AS1*	34 HCC tissues and paired ANTs	HepG2, Hep3b	miR-372-3p, Rab23	–	Patient survival, tumor size, tumor stages	Its knockdown suppresses cell proliferation, invasion and migration and induces apoptosis *via* miR-372-3p-mediated upregulation of Rab23	(161)
*DLEU2*	50 HCC tissues and paired ANTs	SMMC7721, L02, Huh7, HCCLM3	EZH2	–	vascular invasion, tumor stage	Its knockdown represses proliferation, migration and invasion of HCC cells	(162)
*DBH-AS1*	45 HCC tissues and paired ANTs, male BALB/C nude mice	HepG2, SMMC-7721, Hep3B, MHCC97H, SK-Hep1, LO2, QSG7701	–	MAPK signaling pathway	HBsAg, tumor size	Promotes proliferation and survival of HCC cells by activating MAPK signaling pathway	(163)
*DBH-AS1*	46 HCC tissues and paired ANTs	Huh7, PLC, HepG2, Hep3B, LO2	miR-138,	AK/Src/ERK signaling pathway	tumor size, TNM stage, lymph node metastasis	Promotes tumorigenesis of HCC through targeting miR-138 by AK/Src/ERK signaling pathway	(164)
*LINC00152*	BALB/c mic	HCCLM3, HepG2, MHCC97L, SNU449, THLE‐3, LO2	miR-215, CDK13	–	–	Its knockdown inhibits proliferation, migration and invasion and induces apoptosis in HCC cells through regulation of miR-215/CDK13 axis	(165)
*LINC00152*	70 HCC tissues and paired ANTs, male BALB/c mice	Hep3B, HCCLM3, MCC97H, HepG2	miR-139, PIK3CA	PI3K/Akt/mTOR signaling pathway	–	Promotes HCC progression through sponging miR-139 and upregulation of PIK3CA	(166)
*LINC00152*	80 HCC tissues and paired ANTs, male athymic BALB/c nude mice	Huh7, HCCLM3, Hep3B	miR-193a/b-3p, CCND1	–	–	Supports cell cycle transition through sponging miR-193a/b-3p and upregulation of CCND1	(167)
*AFAP1-AS1*	156 HCC tissues and paired ANTs, nude mice	LO2, SMMC-7721, Bel-7402, MHCC-97 L, MHCC-97H	–	–	Patient survival, tumor size, TNM stage, vascular invasion	Its silencing attenuates proliferation, migration and invasion and induces apoptosis in HC cells	(168)
*LNC473*	70 HCC tissues and paired ANTs	Hep3B, Huh-1, SMMC-7721, PLC/PRF/5, SK-Hep-1	survivin	–	tumor size, BCLC stage, vascular invasion	Promotes proliferation, invasion and EMT process and suppresses apoptosis in HCC cells *via* stabilizing survivin	(169)
*CHRF*	48 HCC tissues and paired ANTs	HepG2, Huh‐7	miR-21	PI3K/AKT and Wnt/β-catenin pathways	TNM stage, differentiation, tumors size	Promotes proliferation, cell viability and EMT process in HCC cells through targeting miR-21	(170)
*NORAD*	29 HCC tissues and paired ANTs	SMMC‐7721, Huh7, PLC/PRF/5, Hep3B	miR-202-5p	TGF-β pathway	Patient survival, HbsAg, tumor size	Stimulates proliferation, migration and invasion of HCC cells *via* targeting miR-202-5p	(171)
*lncPARP1*	70 HCC tissues and paired ANTs, male BALB/c nude mice	SMMC-7721, HepG2, Huh7, SK-Hep-1, PLC/PRF/5, Bel-7402	PARP1	–	Patient survival, elder age, serum level of α-fetoprotein (AFP), tumor size, recurrence	Its knockdown suppresses proliferation, migration, and invasion, while induced apoptosis in HCC cells *via* regulating PARP1	(172)
*lncARSR*	92 HCC tissues and paired ANTs, male athymic BALB/c nude mice	SMMC-7721, HepG2	PTEN	PI3K/Akt signaling pathway	Patient survival, tumor size, BCLC stage	Promotes doxorubicin resistance of HCC cells through downregulating PTEN and activation of PI3K/Akt signaling pathway	(173)
*LASP1-AS*	423 HCC tissues and paired ANTs, athymic male BALB/c nude mic	HCCLM, MHCC97H, d PLC/ PRF/5, Hep3B, HepG2, SMMC-7721, Bel‐7402, Huh7	LASP1	–	Patient survival, tumor size, tumor encapsulation, TNM stage	Supports proliferation, migration and invasion of HCC cells *via* upregulation of LASP1	(174)
*CCHE1*	112 HCC tissues and paired ANTs	MHCC97H, HepG2, Hep3B, Huh-7, HCCLM3, L02	–	ERK/MAPK signaling pathway	Patient survival, tumor number, tumor size, TNM stage	Its knockdown induces growth arrest and apoptosis in HCC cells	(175)
*TUC338*	12 HCC tissues and paired ANTs, male nude mice	HepG2, SMMC-7721, BEK-7402, Hep3B, Huh-7	RASAL1	–	–	Its down-regulation constrains cell proliferation and invasion and sensitizes HCC cells to sorafenib by activation of RASAL1.	(176)
*GIHCG*	70 HCC tissues and paired ANTs, male athymic BALB/c nude mice	L02, QSG7701, SMMC7721, Hep3B, Huh7, HCCLM3	miR-200b/a/429	–	Patient survival, tumor size, microvascular invasion, BCLC stage	Stimulates proliferation, migration and invasion of HCC cells *via* epigenetically silencing miR-200b/a/429	(177)
*lncAKHE*	60 HCC tissues and paired ANTs, 10 male BALB/c nude mice	LO2, Hep3B, 7402, Huh7, HepG2	YEATS4	NOTCH2 signaling pathway	Patient survival	Stimulates proliferation and migration of HCC cells *via* cooperating with YEATS4 and activation of NOTCH2 signaling	(178)
*DUXAP10*	32 HCC tissues and paired ANTs	HepG2, SMMC7721, LO2	–	PI3K/Akt and Wnt/β-catenin signaling pathway	–	Its knockdown suppresses proliferation, migration and invasion and induces apoptosis in HCC cells	(179)
*ZEB1-AS1*	102 HCC tissues and 21 healthy liver samples, athymic BALB/C mice	Huh7, HepG2, Hep3B, SMMC7721, LM3, LO2	–	–	Patient survival, microvascular invasion, recurrence	Influences tumor growth and metastasis in HCC cells	(180)
*MYCNOS*	30 HCC tissues and paired ANTs, female BALB/c mice	HL-7702, Huh-7, Hep3B, JHH-7, SNU398	miR-340, PREX2	–	Patient survival	Influences proliferation and invasion of HCC cells through sponging miR-340 and upregulation of PREX2	(181)
*AGAP2-AS1*	137 HCC tissues and paired ANTs	LO2, Hep3B, HCCLM3, Huh7, MHCC-97H, SMMC-7721	miR-16-5p, ANXA11	AKT signaling pathway	Patient survival, TNM stage, venous invasion, Edmondson, tumor size	Promotes proliferation, migration, invasion and EMT process and suppresses apoptosis in HCC cells through sponging miR-16-5p and upregulation of ANXA11	(182)
*Linc00176*	–	HepG2, Huh7, Hep3B, HLE, HLF, HeLa, HEK29	miR-9, miR-185	–	Patient survival	Its knockdown disrupts the cell cycle and activates necroptosis in HCC cells through releasing miR-9 and miR-185	(183)
*AK002107*	134 HCC tissues and paired ANTs, BALB/c nu/nu mice	HepG2, MHCC97H, MHCC97L, SMMC7721, Hep3B, BEL7402, LO2	miR-140-5p, TGFBR1	–	Patient survival, Child-Pugh stage, AFP, macrovascular invasion, microvascular invasion, tumor size	Induces HCC progression and EMT process through regulating miR-140-5p/TGFBR1 axis	(184)
*DDX11-AS1*	40 HCC tissues and paired ANTs, 6 immune-deficient nude mice	(HepG2, SMMC-7721, SK-hep1, Huh7, HCCLM3, LO2	LATS2	–	Patient survival, serum AFP, TNM stage	Promotes HCC progression and metastasis by repressing LATS2 expression	(185)
*GATA3-AS1*	80 HCC tissues and paired ANTs	Hep3B, HCCLM3	PTEN, CDKN1A, TP53	–	Patient survival, tumor size, TNM stage, lymph node metastasis	Promotes proliferation and metastatic ability of HCC cells through repressing PTEN, CDKN1A and TP53	(186)
*DLEU1*	56 HCC tissues and paired ANTs, male BALB/c nude mice	SMMC-7721, Hep3B, HepG2, Huh‐7, LO2	miR-133a, IGF-1R	PI3K/AKT signaling pathway	Patient survival, TNM stage, vascular metastasis	Endorses HCC progression through sponging miR-133a and regulation of IGF-1R	(187)
*Lnc-Myd88*	110 HCC tissues and paired ANTs, BAB/c nude mice	HepG2, SNU423, SMMC-7721, Hep3B, 97H, 97 L, Huh7, L02	Myd88, H3K27Ac	NF-κB and PI3K/AKT signal pathways	Tumor size, metastasis, Edmondson grade	Endorses proliferation and metastasis of HCC cells through increasing Myd88 expression and by H3K27 modification	(188)
*KTN1-AS1*	80 HCC tissues and paired ANTs, mice	Huh7, MHCC97H, SMMC-7721, Bel-7402, LO2	miR-23c, ERBB2IP	–	Patient survival, tumor size, tumor grade TNM stage	Promotes proliferation and tumor growth of HCC by regulating miR-23c/ERBB2IP axis	(189)
*Linc-GALH*	108 HCC tissues and paired ANTs, 12 normal liver tissues	Huh7, SNU-423, MHCC-97H, MHCC-97L, SMMC-7721, Hep3B, HepG2, L02	Gankyrin	–	Patient survival, vascular invasion, intrahepatic metastasis, distant metastasis,	Promotes migration and invasion HCC cells *via* epigenetically regulating Gankyrin	(190)
*MITA1*	SCID mice	HepG2, A549, U87, PC3, Huh7, HCCLM3, SK-Hep1, SMMC-7721, LO2, HGC27, U251	Slug	–	–	Its knockdown suppresses migration and invasion of HCC cells	(191)
*lnc-UCID*	139 HCC tissues and paired ANTs female NSG mice	HEK293T, LO2, HepG2, QGY-7703	CDK6	–	Patient survival	Promotes cell cycle progression and HCC growth through suppressing DHX9-Mediated CDK6 Down-regulation	(192)
*EIF3J-AS1*	80 HCC tissues and paired ANTs	HepG2, SMMC-7721, MHCC97H, MHCC97H, LO2	miR-122-5p, CTNND2		tumor size, vascular invasion, tumor stage	Its knockdown suppresses proliferation, migration and invasion of HCC cells through regulation of miR-122-5p/CTNND2 axis	(193)
*lncRNA n335586*	3 HBV positive HCC tissues and 3 HBV negative HCC tissues, female athymic BALB/c nude mice	Huh7, HepG2	miR-924, CKMT1A	–	–	Promotes migration, invasion and EMT process through sponging miR-924 and upregulation of CKMT1A	(194)
*FGFR3-AS1*	49 HCC tissues and 15 paired peritumor tissues, male BALB/c nude mice	SMMC-7721, BEL-7404 (7404), Huh7, Hep3B, HepG2, HL-7702	–	PI3K/AKT signaling pathway	–	Its knockdown suppresses proliferation, migration and invasion and induces apoptosis in HCC cells	(195)
*LINC00473*	Male nude mice	SMCC-7721, HepG2, Huh-7, HCCLM3, QGY-7703, QSG-7701	miR-195, HMGA2	–	–	Contributes to HCC progression through sponging miR-195 and upregulation of HMGA2	(196)
*LINC01551*	60 HCC tissues and paired ANTs	L‐02, MHCC97-H, HepG2, SMCC7721	miR-122-5p, ADAM10	–	–	Enhances proliferation, migration and invasion of HCC cells *via* sponging miR-122-5p and upregulation of ADAM10	(197)
*lncRNA-6195 (TCONS_00006195)*	47 HBV-related HCC tissues and ANT	Huh7, HepG2, 293T, L02	ENO1	–	Patient survival, Edmondson-Steiner grade	Suppresses proliferation of HCC cells through repressing enzymatic activity of ENO1 and inhibiting the energy metabolism	(198)
*LINC00511*	127 HCC tissues and paired ANTs	LO2, Hep3B, HepG2, SMMC-7721, MHCC97H, Huh7, HCCLM3	miR-424	–	Patient survival, nodal metastasis, vascular invasion, clinical stage	Promotes proliferation and metastasis of HCC cells through modulating miR-424	(199)
*LINC00511*	Expression data of HCC patients obtained from GEO and TCGA	SMCC7721, HepG2, Huh7, Hep3B, L-02	miR-195, EYA1	–	Patient survival, tumor stage	Promotes HCC progression through sponging miR-195 and upregulation of EYA1	(200)
*linc00462*	49 HCC tissues and paired ANTs	HCC-LM3, Huh7, SK-hep-1, QSG-7701	–	PI3K/AKT signaling pathway	portal vein tumor thrombus tumor size, tumor number, BCLC stage	Its down-regulation decreases proliferation, migration and invasion of HCC cells.	(201)
*NR027113*	134 HCC tissues and paired ANTs	Bel-7402, SK-HEP-1, PLC/PRF/5, MHCC97H, SMMC-7721		PI3K/Akt signaling pathway	Patient survival, TNM stage, tumor size	Its down-regulation decreases proliferation, metastasis and EMT process in HCC cells	(202)
*ASLNC02525*	5 HCC tissues and paired ANTs	.HepG2, QGY-7701, SMMC-7721, L-02	hsa-miR-489-3p, twist1	–	–	Its silencing suppresses proliferation and invasion of HCC cells through regulating hsa-miR-489-3p/twist1 axis	(203)
*LncDQ*	84 HCC tissues and paired ANTs, 50 serum samples from HCC patients and 30 serum samples from healthy controls, male BALB/c athymic nude mice	Huh-7, HepG2, HepG3B, SMMC7721, L02	–	–	Patient survival, tumor stage, lymph node metastasis, tumor number	Its down-regulation decreases proliferation, migration and invasion of HCC cells	(204)
*LINC00963*	48 HCC tissues and paired ANTs	L-02, HepG2, HB611, HHCC	–	PI3K/AKT signaling pathway	Patient survival, tumor size, TNM stage	Promotes proliferation of HCC cells through activating PI3K/AKT signaling pathway	(205)
*DCST1-AS1*	60 HCC tissues and paired ANTs, immunodeficient mice	L02, HepG2, SMMC-7721, Bel-7404, SK-hep-1	miR-1254, FAIM2	–	Patient survival, tumor size	Its knockout suppresses proliferation and induces apoptosis and cell cycle arrest through regulating miR-1254/FAIM2 axis	(206)
*lncRNA00673*	55 HCC tissues and paired ANTs, male BALB/c mice	HepG2, Hep3B, MHCC-97H, L02	–	Notch signaling pathway	–	Its knockdown suppresses proliferation and induces cell cycle arrest and apoptosis in HCC cells	(207)
*TGFB2-AS1*	–	HepG2	–	–	Tumor stage	Its down-regulation decreases proliferation, migration and invasion and induces apoptosis in HCC cells	(208)
*FLVCR1-AS1*	60 HCC tissues and paired ANTs, BALB/c nude mice	LO2, Hep3B, HepG2, Huh7, PLC/PRF-5	miR-513c, MET	–	TNM stage, tumor size	Promotes HCC development and progression through sponging miR-513c and upregulation of MET	(209)
*LINC00707*	12 BALB/c mice	SMCC7721, HepG2, Hep3B, SNU-449, Huh7, LO2	miR-206, CDK14	–	–	Promotes HCC progression *via* sponging miR-206 and upregulation of CDK14	(210)
*lncZic2*	12 advanced HCC tissues, 7 early HCC tissues and 19 peritumor specimens, BALB/c nude mice	–	MARCKS, MARCKSL1	–	–	Regulates self-renewal of liver tumor-initiating cells by increasing MARCKS and MARCKSL1 expression through interacting with BRG1	(211)
*GHET1*	68 HCC tissues and paired ANTs	HepG2, Hep3B, Bel-7402, SMMC-7721 HCC, L02	KLF2	–	Patient survival, vascular invasion, cirrhosis, tumor size, edmindson grade	Promotes proliferation of HCC cells through epigenetically silencing KLF2	(15)
*lncRNA 00152*	58 HCC tissues and paired ANTs	MHCC97, Huh7, HB611, LO2	–	JAK2/STAT3 signaling pathway	tumor stage, tumor size	Promotes cell proliferation and cell cycle progression by activating JAK2/STAT3 signaling pathway	(212)
*OR3A4*	78 HCC tissues and paired ANTs	L02, HUVECs, (Huh7, SMMC-7721, HepG2, Hep3B	–	AGGF1/akt/mTOR pathway	Patient survival, tumor size, tumor differentiation, Edmondson Grade, vascular invasion	Its down-regulation decreases proliferation, migration, invasion and angiogenesis in HCC cells.	(213)
*PAPAS*	74 HCC tissues and paired ANTs, plasma samples from 74 HCC patients and 52 healthy controls	SNU-398, SNU‐182	miR-188-5p	–	–	Promotes HCC cells proliferation through interacting with miR-188-5p	(214)
*LINC01433*	12 BALB/c mice	Huh‐7, HepG2, Hep3B, MHCC97L, SMCC-7721, LO2	miR-1301, STAT3	–	–	Promotes proliferation, invasion and colony formation ability through modulating miR-1301/STAT3 axis	(215)
*PITPNA-AS1*	60 HCC tissues and paired ANTs, BALB/c nude mice	HepG2, SMMC-7721, HCCLM3, Hep3B, L02, 293T	miR-876-5p, WNT5A	–	Patient survival, metastasis, TNM stage	Promotes proliferation, migration and EMT process in HCC cells through targeting miR-876-5p and modulating WNT5A expression	(216)
*BC200*	45 HCC tissues and paired ANTs, 18 male BALB/c nude mice	HepG2	c−Myc	–	–	Promotes HCC cells migration but has no significant effect on cell proliferation	(217)
*LINC00470*	80 HCC tissues and paired ANTs	LO2, Hep3B, SK-Hep-1, SMMC-7721, Huh7, PLC/PRF/5, HepG2	NF45/NF90, cyclin E1	–	Patient survival, tumor size, TNM stage	Promotes proliferation of HCC cells *via* interacting with NF45/NF90 and stabilizing cyclin E1	(218)
*CASC15*	42 HCC tissues and paired ANTs, female BALB/c nude mice	HUH7, HCCLM3	miR-33a-5p, TWIST1	–	–	Promotes proliferation, migration and invasion and reduces apoptosis in HCC cells *via* sponging miR-33a-5p and upregulation of TWIST1	(219)
*LINC00460*	60 HCC tissues and paired ANTs, serum samples from 60 patients and 60 healthy controls, 12 BALB/c nude mice	HepG2, Hep3B, SNU-449, THLE-3 cells, HCCLM3, Huh-7, LO2	miR-485-5p, PAK1	–	tumor differentiation grade, tumor dimension, capsular integrity, TNM stage, metastasis	Promotes HCC progression by sponging miR-485-5p and upregulation of PAK1	(220)
*TINCR*	60 HCC tissues and paired ANTs	H1581, SNU-475,	miR-214-5p, ROCK1	–	tumor size, TNM stage	Promotes migration and invasion of HCC cells *via* sponging miR-214-5p and upregulation of ROCK1	(221)
*RHPN1-AS1*	40 HCC tissues and paired ANTs	Hep3B, Huh7, SMMC-7721, MHCC97, Bel-7402, QSG-7701, HEK-293T	miR-596, IGF2BP2	–	Patient survival, lymphatic metastasis, AFP	Promotes proliferation and metastasis and reduces apoptosis by regulating miR-596/IGF2BP2 axis	(222)

### Down-Regulated lncRNAs in HCC

Through a high throughput approach, Ni et al. have identified uc.134 as a novel lncRNA which is under-expressed in a highly aggressive HCC cell line. They further verified its down-regulation in clinical HCC samples compared with paired nearby tissues. Notably, down-regulation of uc.134 has been related with poor prognosis of HCC patients. Functionally, this lncRNA suppresses cell proliferation, invasion, and metastasis through binding with CUL4A suppressing its nuclear export. Besides, uc.134 suppresses the CUL4A-associted ubiquitination of LATS1 and enhances YAPS127 phosphorylation which results in down-regulation of YAP target genes of YAP (223). LncRNA-PRAL has been shown to suppress HCC growth and stimulate apoptosis *via* a p53-dependent route. Certain motifs at the 5’ end of this lncRNA have been identified that participate in competitive inhibition of MDM2-dependent p53 ubiquitination (224). Expression of the lncRNA-LET has been decreased in HCC. Further experiments have shown the role of hypoxia-induced histone deacetylase 3 in down-regulation of this lncRNA. Notably, repression of lncRNA-LET has been identified as an important step in the stabilization of nuclear factor 90 protein and subsequent hypoxia-associated tumor cell invasion. The association between down-regulation of lncRNA-LET and metastatic potential of HCC has also been verified in clinical samples (225). TSLNC8 is also down-regulated in HCC samples. Down-regulation of this lncRNA in HCC has been shown to confer malignant phenotype. TSLNC8 competitively interacts with transketolase and STAT3 and alters the phosphorylation patterns and transcriptional activity of STAT3 leading to suppression of the IL-6-STAT3 signaling (226). CASC2 is another down-regulated lncRNAs in HCC samples, particularly in the samples obtained patients with aggressive and recurrent forms of HCC. CASC2 suppresses migration and invasive properties of HCC cells and inhibits EMT program in these cells. Mechanistically, it serves as a competing endogenous RNA for miR-367 to increase expression of its target gene FBXW7. Notably, CASC2 down-regulation and miR-367 up-regulation have been associated with the metastasis-associated characteristics in the clinical samples (227). **Table 2** displays the impact of down-regulated lncRNAs in HCC.

**Table 2:** List of under-expressed lncRNAs in HCC (ANT, adjacent non-cancerous tissue).

lncRNA	Sample	Assessed cell line	Gene interaction	Signaling pathway	Association with clinical features	Function	Reference
*PSTAR*	127 HCC tissues and ANTs	PHH, HUCPM, HepG2, MHCC-97H, HCCLM3, Hep3B, Huh7, HEK293T, HCT116	p53, hnRNP K	p53 signaling pathway	Patient survival, tumor size, tumor stage	Suppresses proliferation and tumorigenicity of HCC cells by promoting p53 signaling and cell cycle arrest	(228)
*TPTEP1*	32 primary HCC tissues and paired ANTs, 18 male BALB/c nude mice	HepG2, SMMC-7721, QGY-7703, Huh-7, MHCC97h, SNU-449, Sk-hep1, and L02	STAT3	–	–	Represses proliferation, invasion and tumorigenicity of HCC cells through inhibiting STAT3 phosphorylation	(229)
*CASC2*	75 HCC tissues and ANTs, nude mice	MHCC-97L, Hep-3B, HepG2, Huh7, SMMC-7721, MHCC-97H, LO2	miR-367, FBXW7	–	Patient survival, venous infiltration, high Edmondson-Steiner grading, TNM tumor stage	Inhibit migration, invasion and EMT process by sponging miR-367 and upregulation of FBXW7	(227)
*CASC2*	30 HCC tissues and paired ANTs	LO2, HepG2, Hep3B, QSG-7701, SMMC-7721, Huh-7	miR-183	Wnt/β-catenin signaling pathway	–	Represses cell viability, colony formation, migration, and invasion through targeting miR-183	(230)
*CASC2*	50 HCC tissues and paired ANTs	HepG2, HuH7, Hep3B, SMMC7221, Bel7402, LO2	–	MAPK signaling pathway	–	Its overexpression suppresses proliferation, migration and invasion and induces apoptosis in HCC cells	(231)
*CASC2*	80 HCC tissues and paired ANTs	HepG2, SMMC-7721, Hep3B, Huh-7, L02	miR-362-5p	NF-κB signaling pathway	tumor size, differentiation statues	Its overexpression suppresses migration and invasiveness of HCC cells through affecting miR-362-5p.	(232)
*CASC2*	20 HCC tissues and paired ANTs, BALB/c nude mice	HepG2, HuH7	miR-24-3p	–	–	Suppresses cell viability and induces apoptosis in HCC cells *via* regulating miR-24-3p	(233)
*EPB41L4A-AS2*	10 HCC tissues and 10 normal tissues, Neonatal B6C3F1 mice	SMMC-7721, QGY-7703, QSG-7701	miR-301a-5p, FOXL1	–	–	Its upregulation inhibits proliferation, migration and invasion by sponging miR-301a-5p and upregulation of FOXL1	(234)
*LINC00467*	65 HCC tissues and paired ANTs	SMMC-7721, HepG2	miR-9-5a, PPARA	–	metastasis	Its ectopic expression reduces proliferation, migration and invasive features of HCC cells through sponging miR-9-5a and increasing PPARA.	(235)
*lnc-DILC*	195 HCC tissues and paired ANTs, NOD-SCID mice	Huh7, HepG2, CSQT-2	IL-6	JAK2/STAT3 activation	Patient survival	Suppresses liver cancer stem cell expansion through inhibition of autocrine IL-6/STAT3 signaling.	(236)
*lnc-FTX*	129 HCC tissues and paired ANTs,	SMMC-7721, HCCLM3, Hep3B, HepG2, Huh7, 97H, GSG7701	miR-374a, MCM2	Wnt/β-catenin signaling pathway	Patient survival	Suppresses proliferation, invasion and EMT process in HCC cells through physically binding miR-374a and MCM2	(237)
*LINC00472*	109 HCC tissues and 35 ANTs	LO2, HepG2, BEL7404, Hep3B, SMMC-7721, Huh-7	miR-93-5p, PDCD4	–	Patient survival	Its forced expression suppressed cell proliferation, migration and invasion and promotes apoptosis through miR-93-5p/PDCD4 axis	(238)
*FENDRR*	30 HCC tissues and paired ANTs, BALB/c male nude mice	HepG2, Hep3B, LO2	GPC3	–	–	Suppresses proliferation, migration and invasion and induces apoptosis in HCC cells through epigenetically silencing GPC3	(239)
*TSLNC8*	120 HCC tissues and paired ANTs, nude mice	Huh-7, SNU-449, SMMC-7721	STAT3	–	Patient survival	Suppresses cell proliferation and metastasis of HCC cells	(226)
*miR503HG*	93 HCC tissues and paired ANTs	SMMC-7721, Huh7, L02	HNRNPA2B1	NF-κB signaling pathway	Patient survival, tumor recurrence	Represses HCC cells invasion and metastasis through stimulation of HNRNPA2B1 degradation	(151)
*MEG3*	54 HCC tissues and paired ANTs, serum samples from 54 HCC patients and 54 healthy controls	Hep G2, SNU-398, C3A, AML12,	TGF-β1	–	Patient survival, distant tumor metastasis	Its silencing promotes proliferation, migration and invasion in HCC cells through upregulation of TGF-β1	(240)
*MEG3*	30 HCC tissues and paired ANTs	293T, SK-HEP-1, Huh7	miR-9-5p, SOX11	–	TNM stage, metastasis	Its overexpression represses cell growth and promotes apoptosis in HCC cells by sponging miR-9-5p and upregulation of SOX11	(241)
*TSLD8*	108 HCC tissues and paired ANTs	SMMC-7721, Huh7, HepG2, Hep3B, L02, HEK293T	WWOX	–	TNM stages, tumor dimension, metastatic ability, occurrence of cancer embolus	Inhibits migration and cell viability of HCC cells through stabilizing WWOX	(241)
*Lnc00312*	23 HCC tissues and paired ANTs, female SCID mice	HepG2, MKN-74	cyclin B1	–	–	Inhibits cell proliferation and induces apoptosis and cell cycle arrest through downregulation of cyclin B1	(242)
*lncNRON*	215 HCC tissues and paired ANTs, 5 male nude mice	QGY-7703, HepG2, BEL-7404, Hep3B, SMMC-7721, MHCC97, L02	NFAT	–	Patient survival, tumor size, tumor differentiation, Vascular tumor thrombus	Suppresses proliferation, migration and invasion of HC cells	(243)
*PTENP1*	–	Mahlavu	miR-17, miR-19b, miR-20a, PTEN, PHLPP	PI3K/AKT signaling pathway	–	Its overexpression suppresses proliferation, migration and invasion and supports autophagy and apoptosis in HCC cells	(244)
*LIN00607*	159 HCC tissues and paired ANTs, nude mice	MHCC97H, HCCLM3, PLC, Hep3B, HepG2, 7721	p65, p53	–	Patient survival	Its overexpression reduces cell proliferation and induces apoptosis in HCC cells through suppression of p65 transcription	(245)
*AOC4P*	108 HCC tissues and paired ANTs, male BALB/C nude mice	J7, SK-Hep1	Vimentin	–	Patient survival, clinical stage, capsule invasion, vessel invasion	Constrains proliferation and metastasis of HCC cells by increasing Vimentin degradation and inhibition of EMT process	(246)
*AK058003*	50 HCC tissues and paired ANTs, male athymic BALB/c nude mice	HepG2, SK-Hep1, HEK 293T	HuR, γ-synuclein	–	–	Suppresses proliferation and metastasis of HCC cells by interacting with HuR and inhibiting γ-synuclein expression	(247)
*Linc-USP16*	70 HCC tissues and paired ANTs,	MHCC97H, MHCC97L, HepG2, SMMC-7721, LO2, BEL7402	miR-21, miR-590-5p, PTEN	AKT signaling pathway	tumor size, clinical stage, metastasis	Suppresses proliferation and migration of HCC cells through regulation of miR-21/miR-590-5p/PTEN route	(247)
*FER1L4*	35 HCC tissues and paired ANTs, 14 Female athymic BALB/c mice	LO2, Hep3B Huh7, 293T	PTEN	–	–	Suppresses proliferation of HCC cells *via* regulating PTEN	(248)
*FER1L4*	36 HCC tissues and paired ANTs, Female nude (BALB/c-nu) mice	HepG2, Huh7, Hep3B, HCCM3, LO2	miR-106a-5p	–	–	Constrains proliferation, invasion and tumorigenicity of HCC cells *via* targeting miR-106a-5p	(249)
*FER1L4*	31 HCC tissues and paired ANTs	HepG‐2, Hep3b, SMMC‐7721, L‐02	–	PII3K/AKT signaling pathway	–	Its overexpression reduces cell proliferation, migration and invasion and induces apoptosis	(250)
*PANDA*	48 HCC tissues and paired ANTs, immunodeficient mice	HCC LM3, Huh7	–	–	–	Its overexpression enhances proliferation of HCC cells by repressing senescence associated inflammatory factor IL8	(251)
*HHIP-AS1*	60 HCC tissues and paired ANTs	Hep3B, PLC/PRF/5, Huh7, HepG2, MHCC-97 h	HHIP	–	tumor size, metastasis, TNM stage	Constrains proliferation, migration and invasion and induces apoptosis in HCC cells *via* stabilizing HHIP	(252)
*XIST*	40 HCC tissues and paired ANTs	HepG2	miR-155-5p	–	–	Its overexpression inhibits HCC cell growth by targeting miR-155-5p	(253)
*JPX*	40 HCC tissues and paired ANTs	HepG2	XIST	–	–	Its overexpression HCC cell growth through upregulation of v	(253)
*uc.134*	170 paraffin-embedded samples of HCC tissues and ANTs, male BALB/c nude mice	MHCC97, HCCLM3, MHCC97L, Huh7, L02, HepG2, Bel7402	LATS1, CUL4A	–	Patient survival, TNM stage, lymph node metastasis, tumor number, Serum AFP,	Constrains proliferation, invasion and metastasis of HCC cells through suppressing CUL4A-mediated ubiquitination of LATS1	(223)
*C1QTNF1-AS1*	11 HCC tissues and paired ANTs, 12 male BALB/C nude mice	HepG2, Huh7	miR-221-3p, SOCS3	JAK/STAT signaling pathway	–	Its overexpression inhibits proliferation, migration and invasion of HCC cells through targeting miR-221-3p and upregulation of SOCS3	(254)
*GAS8-AS1*	82 HCC tissues and paired ANTs, male nude BALB/c mice	HepG2, SMMC7721	GAS8	–	Patient survival	Suppresses proliferation, migration and invasion and induces apoptosis by epigenetically activating GAS8	(255)
*LINC00657*	49 HCC tissues and paired ANTs, female nude (BALB/c-nu) mice	HepG2, Huh7, Hep3B, Bel-7402, SMMC-7721, HCCM3	miR-106a-5p, PTEN	–	Patient survival, tumor size, vascular invasion, TNM stage	Suppresses proliferation, migration and invasion through sponging miR-106a-5p and regulation of PTEN expression	(256)
*Linc-cdh4-2 (TCONS_00027978)*	–	SK-Hep-1, Huh7	R-cadherin	–	–	Represses migration and invasion of HCC cells through regulation of R-cadherin	(257)
*MAGI2-AS3*	88 HCC tissues and paired ANTs, 12 male BABL/c nude mice	L02, HepG2, Hep3B, MHCC‐97H	miR-374b-5p, SMG1	–	Patient survival, tumor size, lymph node metastasis, TNM stage	Suppresses proliferation and migration of HCC cells *via* sponging miR-374b-5p and increasing SMG1	(258)
*LINC01093*	70 HCC tissues and paired ANTs, BALB/c-nu/nu mice	Huh7, BEL-7402	IGF2BP1, GLI1	–	Patient survival, cancer embolus, TNM stage	Suppresses proliferation and metastasis of HCC cells *via* interaction with IGF2BP1 and facilitation of GLI1 degradation	(259)
*GAS5*	50 HCC tissues and paired ANTs	Huh7, Hep3B, HepG2, QGY-7701, MHCC97L, HCCLM9he, L02	vimentin	–	Patient survival, PVTT, histologic grade	Inhibits proliferation and invasion of HCC cells through regulating Vimentin	(260)
*GAS5*	32 HCC tissues and paired ANTs	Bel-7402, SMMC-7721, HCCLM3, L-02	miR-21	–	Patient survival, TNM stage, tumor size	Its overexpression suppresses migration and invasion of HCC cells through targeting miR-21	(261)
*GAS5*	32 HCC tissues and paired ANTs, mice	HepG2, HepB3, LO2	miR-21, PTEN	–	Patient survival	Its downregulation promotes proliferation and drug resistance HCC cells through reducing PTEN	(262)
*GAS5*	38 HCC tissues and paired ANTs	Lo-2, HepG2, Huh7	miR-222	VEGF signaling pathway	Patient survival	Enhances sensitivity of HCC cells to cisplatin through sponging miR-222	(262)
*SchLAH*	132 HCC tissues and paired ANTs, BALB/c nude mice	HepG2, Hep3B, SMMC7721	FUS	–	Patient survival	Represses migration and lung metastasis of HCC cells *via* interacting with FUS	(263)
*NKILA*	54 HCC tissues and paired ANTs	QSG-7701, SMMC-7721, Hep3B, HCCLM3, HepG2	–	NF-κB signaling	Patient survival	Its overexpression enhances baicalein effect on inhibition of proliferation and migration and induction of apoptosis	(264)
*LINC00261*	66 HCC tissues and paired ANTs	SMCC-7721, MHCC97L, MHCC97H, LO2	–	Notch signaling pathway	Patient survival, tumor size, TNM stage	Inhibits proliferation, colony formation, invasion and EMT process	(265)
*MIR31HG*	42 HCC tissues and paired ANTs, BALB/c nude mice	SMMC7721, HepG2, Huh7, SK-hep1, L02	miR-575, ST7L	–	Patient survival, TNM stage, tumor size, tumor nodule number, vascular invasion	Suppresses proliferation, migration and invasion of HCC cells through sponging miR-575 and regulation of ST7L expression	(266)
*LINC01554*	167 HCC tissues and paired ANTs	BEL7402, QGY7701, QGY7703, SMMC7721, PLC8024, HepG2, Huh7, Hep3B	miR-365a, PKM2	Akt/mTOR signaling pathway	Patient survival, tumor invasion, tumor size, tumor stage	Inhibits cell growth, colony formation in soft agar, foci formation, and tumor formation through downregulation of PKM2	(267)
*FAM99B*	80 HCC tissues and paired ANTs	MHCC97L, MHCC97H, HCCLM3, Huh-7, HepG2, Hep3B	–	–	Patient survival, vascular invasion, histologic grade, T stage	Its overexpression suppresses proliferation, migration and invasion of HCC cells	(268)
*RGMB-AS1*	108 HCC tissues and 25 ANTs	QGY-7703, HuH7, BEL7402, HepG2	RGMB	–	Patient survival, clinical stage, tumor size, metastasis	Its overexpression represses proliferation, migration and invasion of HCC cells	(269)
*LINC00052*	12 HCC tissues and paired ANTs	SMMC7721, HepG2, SK-hep1, Huh7, L02, 293T	miR-101-3p, SOX9	–	–	Constrains proliferation and metastasis *via* affecting miR-101-3p and suppressing SOX9	(270)
*DGCR5*	–	HepG2, Hep3B, MHCC-97L, SNU-449, MHCC-97H, SMCC7721, THLE-3	miR-346, KLF14	–	–	Its overexpression attenuates proliferation, migration and invasion of HCC cells through sponging miR-346 and modulating KLF14 expression	(271)
*ID2-AS1*	144 HCC tissues and paired ANTs, NOD-SCID mice	MHCC97L, MHCC97H, HCCLM3, Huh7, HepG2-C3A, SK-Hep1, HEK-293T	ID2	–	Patient survival	Represses migration, invasion and metastasis of HCC cells *via* binding to HDAC8 and regulation of ID2 expression	(272)
*F11-AS1*	–	HepG2, Hep3B, Huh-6, SMMC7721, LO2	miR-3146, PTEN	–	–	Represses HCC progression *via* acting as ceRNA for miR-3146 and affecting PTEN level	(273)

## Diagnostic and Prognostic Impact of lncRNAs in HCC

Expression patterns of several lncRNAs have been related with overall survival or disease-free survival of patients with liver neoplasm. Oncogenic lncRNAs which decrease survival of HCC patients include NEAT1, PTTG3P, UBE2CP3, LINC00461, MALAT1, MNX1-AS1, MCM3AP-AS1, ANRIL, AWPPH, PVT1, SNHG1, ENST00000429227.1, LINC00665, CRNDE, FOXD2-AS1, HULC and some other lncRNAs. Instead, low expressions of several tumor suppressor lncRNAs namely PSTAR, CASC2, lnc-FTX, LINC00472, TSLNC8, miR503HG, MEG3, LIN00607, AOC4P, uc.134, GAS8-AS1, LINC00657, MAGI2-AS3, LINC01093, GAS5, SchLAH, and NKILA predict patients’ outcome. Univariate/multivariate cox regression analyses have confirmed the role of these lncRNAs in the determination of HCC prognosis. **Table 3** lists the results of studies which evaluated the prognostic roles of lncRNAs in patients with HCC.

**Table 3:** Prognostic role of lncRNAs in HCC (ANT, adjacent non-cancerous tissue; OS, overall survival; RFS, relapse-free survival; DFS, disease-free survival; PFS, progression-free survival; TTR, time to tumor recurrence).

lncRNA	Sample number	Kaplan-Meier analysis	Univariate cox regression	Multivariate cox regression	Reference
*NEAT1*	40 HCC specimens and paired ANTs	Its elevated level is related with short OS.	–	–	(24)
*NEAT1*	86 HCC specimens and paired ANTs	Its elevated level is related with poor OS.	correlated with OS	an independent prognostic factor for OS	(27)
*PTTG3P*	90 paraffin-embedded HCC specimens and ANTs	Its elevated level is related with low OS.	–	an independent prognostic factor for OS	(31)
*UBE2CP3*	46 HCC specimens and ANTs	Its elevated level is related with poor OS.	–	–	(33)
*LINC00461*	87 HCC specimens and paired ANTs	Its elevated level is related with decreased OS.	–	–	(34)
*MALAT1*	56 HCC specimens and paired ANTs	Its elevated level is related with decreased OS.	–	–	(35)
*MNX1-AS1*	81 HCC specimens and paired ANTs	Its elevated level is related with poor OS.	–	–	(37)
*MCM3AP-AS1*	80 HCC specimens and paired ANTs	Its elevated level is related with shorter OS.	–	–	(38)
*ANRIL*	130 tissues and paired ANTs	Its elevated level is related with low OS.	correlated with OS	an independent prognostic marker for OS	(49)
*AWPPH*	88 HCC specimens and paired ANT	Its elevated level is related with poor DFS and OS.	–	an independent prognostic factor for RFS and OS	(51)
*PVT1*	48 HCC specimens and paired ANTs	Its elevated level is related with poor OS.	–	–	(53)
*SNHG1*	82 HCC specimens and paired ANTs	Its elevated level is related with poor RFS and OS.	–	–	(56)
*ENST00000429227.1*	161 HCC specimens and paired ANTs	Its elevated level is related with poor OS.	correlated with OS	an independent prognostic marker for OS	(58)
*LINC00665*	76 HCC specimens and paired ANTs	Its elevated level is related with shorter OS	–	–	(63)
*CRNDE*	23 HCC specimens and paired ANTs	Its elevated level is related with shorter DFS and OS.	–	–	(65)
*FOXD2-AS1*	88 HCC specimens and paired ANTs	Its elevated level is related with poor OS.	–	–	(70)
*HULC*	41 HCC specimens and paired ANTs	Its elevated level is related with shorter OS.	correlated with OS	Its expression pattern is not an independent prognostic marker for PFS and OS.	(76)
*SBF2-AS1*	134 HCC specimens and paired ANTs	Its elevated level is related with shorter OS.	correlated with OS	Its expression pattern is not an independent prognostic marker for OS.	(78)
*UC001kfo*	82 HCC tissues and 20 ANTs	Its elevated level is related with poor progression-free survival (PFS) and OS.	correlated with PFS and OS.	an independent prognostic marker for PFS and OS	(79)
*LUCAT1*	90 HCC tissues and paired ANTs	Its elevated level is related with poor OS.	correlated with OS	an independent prognostic marker for OS	(82)
*AK001796*	73 HCC tissues and paired ANTs	Its elevated level is related with poor OS.	–	an independent prognostic marker for OS	(83)
*FEZF1-AS1*	139 HCC tissues and paired ANTs	Its elevated level is related with poor OS.	–	–	(84)
*MINCR*	161 HCC tissues and paired ANTs	Its elevated level is related with poor OS.	correlated with OS	an independent prognostic marker for OS	(85)
*XIST*	88 HCC tissues and paired ANTs	Its elevated level is related with short DFS.	–	–	(88)
*XIST*	52 HCC tissues and paired ANTs	Its elevated level is related with poor survival of HCC patients.	–	–	(89)
*TRPM2-AS*	108 HCC tissues and paired ANTs	Its elevated level is related with poor OS.	–	–	(90)
*LSINCT5*	126 HCC tissues and paired ANTs	Its elevated level is related with poor OS.	–	–	(92)
*XLOC*	68 HCC tissues and paired ANTs	Its elevated level is related with poor OS.	–	an independent prognostic marker for OS	(93)
*URHC*	52 HCC tissues and paired ANTs	Its elevated level is related with short OS after surgery.	–	–	(96)
*RUSC1-AS-N*	66 HCC tissues and paired ANTs	Its elevated level is related with short RFS and OS.	–	–	(99)
*CCAT1*	66 HCC tissues and paired ANTs	Its elevated level is related with low RFS and OS.	–	–	(101)
*SNHG16*	71 HCC tissues and paired ANTs	Its elevated level is related with poor DFS and OS.	correlated with OS	an independent prognostic marker for OS	(105)
*SNHG12*	48 HCC tissues and paired ANTs	Its elevated level is related with poor RFS and OS.	–	–	(108)
*SNHG20*	96 HCC tissues and paired ANTs	Its elevated level is related with poor OS.	–	–	(109)
*SNHG5*	48 HCC tissues and paired ANTs	Its elevated level is related with poor RFS and OS.	correlated with RFS and OS	an independent prognostic marker for RFS and OS	(110)
*SNHG6-003*	52 HCC tissues and paired ANTs, FFPE tissues from 160 patients	Its elevated level is related with poor DFS and OS.	correlated with OS	an independent prognostic marker for OS	(113)
*SNHG7*	40 HCC tissues and paired ANTs	Its elevated level is related with low OS.	–	–	(114)
*SNHG7*	80 HCC tissues and paired ANTs	Its elevated level is related with short OS.	–	–	(115)
*Sox2ot*	84 HCC tissues and ANTs	Its elevated level is related with poor OS.	correlated with OS	an independent prognostic marker for OS	(119)
*SPRY4-IT1*	82 HCC tissues and paired ANTs	Its elevated level is related with poor OS.	–	–	(121)
*PANDAR*	482 HCC tissues and paired ANTs	Its elevated level is related with poor OS.	correlated with OS	an independent prognostic marker for OS	(122)
*CARLo-5*	97 HCC tissues and paired ANTs	Its elevated level is related with shorter DFS and OS.	correlated with DFS and OS	an independent risk factor for DFS and OS	(124)
*PlncRNA-1*	84 HCC tissues and paired ANTs	Its elevated level is related with poor OS.	correlated with OS	an independent prognostic factor for OS	(126)
*PCAT-14*	39 HCC tissues and paired ANTs	Its elevated level is related with poor OS.	correlated with OS	an independent prognostic factor for OS	(129)
*DLX6-AS1*	60 HCC tissues and paired ANTs	Its elevated level is related with poor OS.		–	(131)
*TP73-AS1*	84 HCC tissues and paired ANTs	Its elevated level is related with poor OS.	correlated with OS	an independent prognostic factor for OS	(138)
*HANR*	35 HCC tissues and paired ANTs,	Its elevated level is related with poor OS.	–	–	(140)
*lncRNA FAL1*	30 HCC tissues and paired ANTs	Its elevated level is related with poor OS.	–	–	(143)
*CDKN2B-AS1*	100 HCC tissues and paired ANTs	Its elevated level is related with poor OS.	–	–	(144)
*lncRNA-PDPK2P*	60 HCC tissues and paired ANTs,	Its elevated level is related with poor OS.	correlated with OS	an independent prognostic factor for OS	(147)
*SOX9-AS1*	67 HCC tissues and paired ANTs	Its elevated level is related with low OS.	–	–	(150)
*SOX21-AS1*	68 HCC tissues and paired ANTs	Its elevated level is related with shorter OS.	–	an independent prognostic factor for OS	(151)
*HOXA11-AS*	66 HCC tissues and paired ANTs	Its elevated level is related with shorter OS.	–	–	(152)
*lncRNA-ATB*	72 HCC tissues and paired ANTs	Its elevated level is related with low OS.	–	–	(156)
*OSER1-AS1*	34 HCC tissues and paired ANTs	Its elevated level is related with shorter DFS and OS.	–	–	(161)
*AFAP1-AS1*	156 HCC tissues and paired ANTs	Its elevated level is related with shorter DFS and OS.	–	–	(168)
*LNC473*	70 HCC tissues and paired ANTs	Its elevated level is related with low OS	–	–	(169)
*NORAD*	29 HCC tissues and paired ANTs	Its elevated level is related with shorter DFS and OS	correlated with OS	an independent prognostic factor for OS	(171)
*lncPARP1*	70 HCC tissues and paired ANTs	Its elevated level is related with shorter DFS and OS.	–	–	(172)
*lncARSR*	92 HCC tissues and paired ANTs	Its elevated level is related with shorter RFS and OS.	–	–	(173)
*LASP1-AS*	423 HCC tissues and paired ANTs	Its elevated level is related with poor RFS and OS.	correlated with RFS and OS	an independent prognostic factor for RFS and OS	(174)
*CCHE1*	112 HCC tissues and paired ANTs	Its elevated level is related with low OS.	correlated with OS	an independent prognostic factor for OS	(175)
*GIHCG*	70 HCC tissues and paired ANTs	Its elevated level is related with low RFS and OS.	–	–	(177)
*lncAKHE*	60 HCC tissues and paired ANTs	Its elevated level is related with low DFS and OS.	–	–	(178)
*ZEB1-AS1*	102 HCC tissues and 21 healthy liver samples	Its elevated level is related with low RFS and OS.	–	an independent prognostic factor for survival	(180)
*MYCNOS*	30 HCC tissues and paired ANTs	Its elevated level is related with poor OS.	–	–	(181)
*AGAP2-AS1*	137 HCC tissues and paired ANTs	Its elevated level is related with poor DFS and OS.	–	–	(182)
*AK002107*	134 HCC tissues and paired ANTs	Its elevated level is related with poor DFS and OS.	–	an independent prognostic factor for DFS and OS	(184)
*DDX11-AS1*	40 HCC tissues and paired ANTs	Its elevated level is related with poor OS.	–	–	(185)
*GATA3-AS1*	80 HCC tissues and paired ANTs	Its elevated level is related with low OS.	–	–	(186)
*DLEU1*	56 HCC tissues and paired ANTs	Its elevated level is related with low OS.	–	–	(187)
*KTN1-AS1*	80 HCC tissues and paired ANTs	Its elevated level is related with low OS.	–	–	(189)
*Linc-GALH*	108 HCC tissues and paired ANTs, 12 normal liver tissues	Its elevated level is related with poor RFS and OS.	–	–	(190)
*LINC00511*	127 HCC tissues and paired ANTs	Its elevated level is related with low OS	correlated with OS	an independent prognostic factor for OS	(199)
*NR027113*	134 HCC tissues and paired ANTs	Its elevated level is related with poor DFS and OS.	–	an independent prognostic factor for survival	(202)
*LncDQ*	84 HCC tissues and paired ANTs, 50 serum samples from HCC patients and 30 serum samples from healthy controls	Its elevated level is related with low OS.	correlated with OS	an independent prognostic factor for OS	(204)
*GHET1*	68 HCC tissues and paired ANTs	Its elevated level is related with low OS.	–	an independent prognostic factor for OS	(15)
*OR3A4*	78 HCC tissues and paired ANTs	Its elevated level is related with poor OS.	–	an independent prognostic factor for HCC	(213)
*PITPNA-AS1*	60 HCC tissues and paired ANTs	Its elevated level is related with poor OS.	–	–	(216)
*AK021443*	193 HCC tissues and paired ANTs	Its elevated level is related with low OS.	correlated with OS	an independent prognostic factor for OS	(274)
*UCA1*	Serum samples from 105 HCC patients, 105 persons with benign liver diseases and 105 healthy controls	Its elevated level is related with low OS.	correlated with prognosis	an independent prognostic factor for HCC	(275)
*SNHG15*	105 HCC tissues and paired ANTs	Its elevated level is related with low OS.	correlated with OS	an independent prognostic factor for OS	(276)
*PSTAR*	127 HCC tissues and ANTs	Its low expression is related with poor OS and RFS.	–	an independent prognostic factor for OS and RFS	(228)
*CASC2*	75 HCC tissues and ANTs	Its low expression is related with poor OS and DFS.	–	–	(227)
*lnc-FTX*	129 HCC tissues and paired ANTs	Its low expression is related with poor OS and RFS.	–	–	(237)
*LINC00472*	109 HCC tissues and 35 ANTs	Its expression is correlated with short OS.	–	–	(238)
*TSLNC8*	120 HCC tissues and paired ANTs	Its low expression is related with low OS.	–	–	(226)
*miR503HG*	93 HCC tissues and paired ANTs	Its expression level is related with TTR and OS.	correlated with TTR and OS	an independent prognostic factor for TTR and OS	(151)
*MEG3*	serum samples from 54 HCC patients and 54 healthy controls	Its low expression is related with shorter survival time.	–	–	(240)
*LIN00607*	159 HCC tissues and paired ANTs	Its low expression is related with low OS.	–	–	(245)
*AOC4P*	108 HCC tissues and paired ANTs	Its low expression is related with low DFS and OS.	–	an independent prognostic factor for DFS and OS	(246)
*uc.134*	170 paraffin-embedded samples of HCC tissues and ANTs	Its low expression is related with low OS.	–	–	(223)
*GAS8-AS1*	82 HCC tissues and paired ANTs	Its low expression is related with poor OS.	–	–	(255)
*LINC00657*	49 HCC tissues and paired ANTs	Its low expression is related with poor OS.	–	–	(256)
*MAGI2-AS3*	88 HCC tissues and paired ANTs	Its low expression is related with poor OS.	–	an independent prognostic factor for OS	(258)
*LINC01093*	70 HCC tissues and paired ANTs	Its low expression is related with short OS.	correlated with OS	an independent prognostic marker for OS	(259)
*GAS5*	50 HCC tissues and paired ANTs	Its low expression is related with short OS.	correlated with OS	an independent prognostic marker for OS	(260)
*GAS5*	71 HCC tissues and paired ANTs	Its low expression is related with short OS.	correlated with OS	an independent prognostic marker for OS	(277)
*GAS5*	38 HCC tissues and paired ANTs	Its low expression is related with short OS.	–	–	(262)
*SchLAH*	132 HCC tissues and paired ANTs	Its low expression is related with poor OS.	–	–	(263)
*NKILA*	54 HCC tissues and paired ANTs	Its low expression is related with poor OS.	–	–	(264)
*LINC00261*	66 HCC tissues and paired ANTs	Its low expression is related with poor OS.	–	–	(265)
*MIR31HG*	42 HCC tissues and paired ANTs	Its low expression is related with poor OS.	–	–	(266)
*LINC01554*	167 HCC tissues and paired ANTs	Its low expression is related with poor OS.	correlated with OS	an independent prognostic factor for OS	(267)
*RGMB-AS1*	108 HCC tissues and 25 ANTs	Its low expression is related with poor OS.	correlated with OS	an independent prognostic factor for OS	(269)
*ID2-AS1*	144 HCC tissues and paired ANTs	Its low expression is related with poor OS.	correlated with OS	an independent prognostic factor for OS	(272)
*CCAT2*	122 HCC tissues and paired ANTs	Its elevated level is related with low OS.	–	an independent prognostic factor for OS	(278)
*GAS5-AS1*	83 HCC tissues and paired ANTs	Its low expression is related with low OS.	correlated with OS	an independent prognostic factor for OS	(279)
*JPX*	68 HCC tissues and paired ANTs, plasma samples from 42 patients and 68 healthy controls	Its low expression is related with low OS.	correlated with OS	an independent prognostic factor for OS	(280)
*XIST*	68 HCC tissues and paired ANTs, plasma samples from 42 patients and 68 healthy controls	Its low expression is related with low OS.	correlated with OS	an independent prognostic factor for OS	
*GMDS-DT*	198 HCC tissues and paired ANTs	Its low expression is related with low DFS and OS.	–	an independent prognostic factor for DFS and OS	(281)
*X91348*	107 HCC tissues and paired ANTs, serum samples from 107 HCC patien6ts and 82 healthy controls	Its low expression is related with low OS.	–	an independent prognostic factor for OS	(282)
*TCONS_00027978*	241 HCC tissues and paired ANTs	Its low expression is related with low DFS and OS.	–	an independent prognostic factor for DFS and OS	(283)

Expression levels of lncRNAs can differentiate HCC tissues from non-tumoral tissues indicating the role of these transcripts as diagnostic biomarkers for HCC. The best diagnostic power values have been reported for NEAT1, PANDAR, CCHE1 and SNHG1. Most notably, serum or plasma levels of a number of lncRNAs such as LINC-ITGB1, LINC00978, LncDQ, PAPAS, MEG3, UCA1 and NEAT1 could be used as diagnostic markers for this kind of cancer (**Table 4**).

**Table 4:** Diagnostic role of lncRNAs in HCC.

lncRNA	Expression pattern	Sample	Type of biomarker	ROC curve analysis			Reference
				Sensitivity	Specificity	Area under ROC curves (AUC)	
*MALAT1*	Upregulated	Tissue samples	Diagnostic biomarker	–	–	0.76	(21)
*LINC-ITGB1*	Upregulated	Serum samples	Diagnostic biomarker (diagnosis of HCC from controls)	–	–	0.8520	(91)
*PANDAR*	Upregulated	Tissue samples	Diagnostic biomarker (diagnosis of HCC)	–	–	0.9564	(122)
*LINC00978*	Upregulated	Serum samples	Diagnostic biomarker (diagnosis of HCC)	76%	96%	0.910	(155)
*CCHE1*	Upregulated	Tissue sample	Diagnostic biomarker (diagnosis of HCC)	–	–	0.9262	(175)
*LncDQ*	Upregulated	Serum samples	Diagnostic biomarker (diagnosis of HCC)	72%	80%	0.804	(204)
*LINC00963*	Upregulated	Tissue samples	Diagnostic biomarker (diagnosis of HCC)	–	–	0.763	(205)
*PAPAS*	Upregulated	Plasma samples	Diagnostic biomarker (diagnosis of Stage I HCC patients from healthy controls)	–	–	0.88	(214)
*MEG3*	Downregulated	Serum samples	Diagnostic biomarker (diagnosis of HCC)	–	–	0.8865	(240)
*FAM99B*	Downregulated	Tissue samples	Diagnostic biomarker (diagnosis HCC from controls)	70.0%	63.7%	0.707	(268)
*UCA1*	Upregulated	Serum samples	Diagnostic biomarker (discriminating HCC patients from healthy controls)	73.3%	99.0%	0.902	(275)
			Diagnostic biomarker (discriminating HCC patients from benign liver disease patients)	71.4%	94.3%	0.848	
*JPX*	Downregulated	Plasma samples	Diagnostic biomarker (diagnosis of HCC)	100%	52.4%	0.814	(280)
*X91348*	Downregulated	Serum samples	Diagnostic biomarker (diagnosis of HCC)	82%	75.4%	0.807	(282)
*MSC-AS1* *POLR2J4* *EIF3J-AS1* *SERHL* *RMST* *PVT1*	Upregulated Upregulated Upregulated Upregulated Upregulated Upregulated	Tissue samples	Diagnostic biomarker (tumor vs. non-tumor)	–	–	0.932	(284)
*CASC2* *TUG1*	Downregulated Upregulated	Blood samples	Diagnostic biomarker (detection of HCC/HCV from HCV and healthy control group)	96.6%	72.5%	–	(285)
*CASC2*	Downregulated	Blood samples	Diagnostic biomarker (detection of HCC/HCV from HCV and healthy control group)	67%	78%	–	
*TUG1*	Upregulated	Blood samples	Diagnostic biomarker (detection of HCC/HCV from HCV and healthy control group)	93.3%	100%	–	
*AC015908.3* *AC091057.3* *TMCC1-AS1* *DCST1-AS1* *FOXD2-AS1*	– – – – –	Tissue samples	Prognostic biomarker (for OS)	–	–	0.769	(286)
*NEAT1*	Upregulated	Serum samples	Diagnostic biomarker (diagnosis HCC from controls)	100%,	88.9%	0.981	(287)
*NEAT1*	Upregulated	Tissue samples	Diagnostic biomarker (diagnosis HCC from controls)	–	–	0.594	(288)
			Prognostic biomarker (prediction of capsule or infiltration)	–	–	0.687	
			Prognostic biomarker (prediction of tumor node)	–	–	0.629	
			Prognostic biomarker (metastasis)	–	–	0.73	
			Prognostic biomarker (portal vein tumor embolus)	–	–	0.656	
			Prognostic biomarker (vaso-invasion)	–	–	0.703	
*GAS5-AS1*	Downregulated	Tissue samples	Diagnostic biomarker (distinguishing HCC from the cirrhosis)	–	–	0.824	(279)
*RP11-160H22.5* *XLOC_014172* *LOC149086*	Upregulated Upregulated Upregulated	Plasma samples	Diagnostic biomarker (diagnosis of HCC)	–	–	0.896	(289)
			Prognostic biomarker (prediction of metastasis)	–	–	0.934	
*Risk score:* *MIR100HG* *SERHL* *CTD-2574D22.4* *SNHG20*	– – – – –	Tissue samples (sequencing data downloaded from TCGA)	Prognostic biomarkers (for OS)	–	–	0.73	(290)
*ENSG00000258332.1*	Upregulated	Serum exosomes	Diagnostic biomarker (discrimination of HCC from chronic hepatitis B)	–	–	0.719	(291)
*LINC00635*	Upregulated	Serum exosomes	Diagnostic biomarker (discrimination of HCC from chronic hepatitis B)	–	–	0.750	
*ENSG00000258332.1* *LINC00635* Along with serum AFP	Upregulated Upregulated – –	Serum exosomes	Diagnostic biomarker (discrimination of HCC from chronic hepatitis B)	–	–	0.894	
*lncRNA-D16366*	Downregulated	Serum samples	Diagnostic biomarker (diagnosis of HCC)	65.5%	84.6%	0.752	(292)
*lncRNA-TSIX*	Upregulated	Serum samples	Diagnostic biomarker (diagnosis of HCC)	87.7%t	72.7%	0.866	(293)
*CASC9*	Upregulated	Serum samples	Diagnostic biomarker (diagnosis of HCC)	–	–	0.933	(294)
*ZFAS1*	Upregulated	Plasma samples	Diagnostic biomarker (diagnosis of HCC)	–	–	0.801	(295)
*lncRNA p34822*	Upregulated	Plasma samples	Diagnostic biomarker (diagnosis of HCC)	80.9%	75.8%	0.845	(296)
*Lnc-PCDH9-13:1*	Upregulated	Salivary samples	Diagnostic biomarker (diagnosis of HCC from healthy controls)	85%	98%	0.898	(297)
			Diagnostic biomarker (diagnosis of HCC from inactive HBsAg carriers)	87%	98%	0.897	
			Diagnostic biomarker (diagnosis of HCC from chronic hepatitis B patients)	87%	98%	0.896	
			Diagnostic biomarker (diagnosis of HCC from liver cirrhosis patients)	87%	92%	0.881	
*SNHG18*	Downregulated	Plasma samples	Diagnostic biomarker (diagnosis of HCC from healthy controls with α-fetoprotein levels below 200 ng/m)	75.61%	73.49%	–	(298)
*SNHG1*	Upregulated	Plasma samples	Diagnostic biomarker (diagnosis of HCC from healthy controls)	–	–	0.92	(299)
*CTC-297N7.9*	Downregulated	Tissue samples	Diagnostic biomarker (diagnosis of HCC)	–	–	0.73	(300)
*LncRNA-AF085935*	Upregulated	Serum samples	Diagnostic biomarker (discrimination of HBV -positive HCC from healthy controls)	–	–	0.988	(301)
			Diagnostic biomarker (discrimination of HBV patients from healthy controls)	–	–	0.664	
			Diagnostic biomarker (discrimination of HBV-positive HCC from HBV patients)	–	–	0.955	
*lncRNA-uc003wbd*	Upregulated	Serum samples	Diagnostic biomarker (discrimination of HBV -positive HCC from healthy controls)	–	–	0.994	
			Diagnostic biomarker (discrimination of HBV patients from healthy controls)	–	–	0.982	
			Diagnostic biomarker (discrimination of HBV-positive HCC from HBV patients)	–	–	0.810	

## Genomic Variants Within lncRNAs and Risk of HCC

Genetic polymorphisms include at least four type of variations namely, single nucleotide polymorphisms, small insertion/deletion polymorphisms, polymorphic repetitive elements and microsatellites. The importance of somatic copy number variations (SCNVs) loci in non-coding regions in the development of HCC has been assessed by Zhou et al. Such investigation has led to identification of recurrent deletion of lncRNA-PRAL in HCC samples in association with poor clinical outcome (224). The lncRNA TSLNC8 on 8p12 is another tumor suppressor lncRNA which is commonly deleted in HCC tissues (226). **Table 5** shows the summarized results of studies which assessed association between lncRNAs insertion/deletion or tetranucleotide repeat polymorphisms and HCC.

**Table 5:** Association between lncRNAs polymorphisms and HCC.

lncRNA	Polymorphism type	Identifier	Samples	Association with HCC	Association with patient outcome	Functional experiments	Reference
*GAS5*	Indel polymorphism	rs145204276	1034 HCC patients and 1054 controls	Deletion allele is associated with increased risk of HCC.	Deletion allele is correlated with higher expression of GAS5 in HCC tissues.	Genotypes of this polymorphism are associated with methylation status of GAS5 promoter region.	(302)
*KCNQ1OT1*	Tetranucleotide repeat polymorphism (STR)	rs35622507	510 HCC patients and 1014 age and sex matched healthy controls	Heterozygote subjects with one allele 10 and those without allele 10 compared with subjects with homozygote 10–10 genotype have decreased risk of HCC.	–	Cell lines without allele 10 have higher expression of KCNQ1OT1.	(303)

## Discussion

LncRNAs contribute in the pathogenesis of HCC through diverse mechanisms including modulation of oncogenes and tumor suppressor genes as well as modification of tumor microenvironment. The latter route of action has been best exemplified by the lnc-EGFR which enhances differentiation of Tregs therefore increasing immune evasion (12). Moreover, certain lncRNAs such as MUF and SNHG7 facilitate EMT process through modulation of Wnt/β-catenin signaling pathway (14, 114). Other lncRNAs can modulate EMT through sponging a number of miRNAs. MAPK, PI3K/AKT and JAK/STAT signaling pathways are other cancer-related pathways that are modulated by several lncRNAs in HCC. The interactions between lncRNAs, miRNAs and mRNAs have functional importance in the pathogenesis of HCC. Examples of such trios include H19/miR-15b/CDC42, H19/miR-326/TWIST1, NEAT1/miR-485/STAT3, MALAT1/miR-124-3p/Slug, MALAT1/miR-195/EGFR, MALAT1/miR-22/SNAI1 and ANRIL/miR-144/PBX3.

Functional roles of lncRNAs in HCC have been appraised in animal models. These models have facilitated identification of lncRNAs targets and related pathways (304), which can be used as therapeutic candidates in HCC. HCC-associated lncRNAs can affect gene expression *via* recruiting epigenetic factors (305), regulation of transcription factors (306), modulation of protein degradation (307) and alteration of phosphorylation of proteins (308).

Genomic alterations and polymorphisms within lncRNA-coding regions have been shown to confer risk of HCC. Such variations might also predict survival of these patients. However, the observed association between these variants and HCC should be verified in independent samples from different ethnic groups. Integration of the results of genome-wide association studies with high throughput sequencing data obtained from microarray and RNA seq experiments would help in discovery of HCC-related single nucleotide polymorphisms within lncRNAs.

The biomarker role of lncRNAs in HCC has been verified by several studies indicating their importance both in the diagnosis and in the prognosis of this cancer. Expression levels of lncRNAs can differentiate HCC patients from inactive HBs Ag carriers, patients with chronic hepatitis and those with liver cirrhosis. In addition, the high diagnostic power values of peripheral levels of a number of lncRNAs such as UCA1 and NEAT1 have potentiated them as methods for non-invasive diagnosis of HCC. Moreover, lncRNAs can be regarded as therapeutic targets in HCC. The importance of lncRNAs as therapeutic targets in HCC has been noted by several experiments in animal models of HCC. Yet, such experiments wait approval in clinical settings. In vivo delivery of a number of lncRNAs such as lncRNA-PRAN, uc.134 and TSLNC8 has been shown to attenuate tumor growth and enhance lifespan of xenograft models of HCC (223, 224, 226). Moreover, a number of lncRNAs such as HULC confer resistance to chemotherapeutic agents (13), indicating the potential of targeted therapies against these transcripts in enhancement of response of HCC patients to conventional therapeutic options. Antisense oligonucleotides and small interfering RNAs are putative methods for suppression of expression of lncRNAs (309, 310) whose efficacies have been verified in animal models and cell line experiments. Yet, this knowledge has not been translated into clinical practice.

Taken together, lncRNAs as important class of regulatory transcripts can influence pathogenesis of HCC from different aspects and can be used as suitable markers for differentiation of HCC from related pathogenic conditions.

## Author Contributions

SG-F and MT wrote the draft and revised it. BH and MG designed the tables and figures. All authors contributed to the article and approved the submitted version.

## Conflict of Interest

The authors declare that the research was conducted in the absence of any commercial or financial relationships that could be construed as a potential conflict of interest.
